# RapD Is a Multimeric Calcium-Binding Protein That Interacts With the *Rhizobium leguminosarum* Biofilm Exopolysaccharide, Influencing the Polymer Lengths

**DOI:** 10.3389/fmicb.2022.895526

**Published:** 2022-07-06

**Authors:** Julián Tarsitano, Lila Y. Ramis, Leonardo G. Alonso, Daniela M. Russo, Angeles Zorreguieta

**Affiliations:** ^1^Fundación Instituto Leloir, Instituto de Investigaciones Bioquímicas de Buenos Aires, Consejo Nacional de Investigaciones Científicas y Técnicas, Buenos Aires, Argentina; ^2^Instituto de Nanobiotecnología (NANOBIOTEC), Consejo Nacional de Investigaciones Científicas y Técnicas-Universidad de Buenos Aires, Buenos Aires, Argentina; ^3^Departamento de Química Biológica, Facultad de Ciencias Exactas y Naturales, Universidad de Buenos Aires, Buenos Aires, Argentina

**Keywords:** cadherin-like domain, exopolysaccharide, biofilm, calcium binding protein, *Rhizobium leguminosarum*, lectin, extracellular matrix, type I secretion system (TISS)

## Abstract

*Rhizobium leguminosarum* synthesizes an acidic polysaccharide mostly secreted to the extracellular medium, known as exopolysaccharide (EPS) and partially retained on the bacterial surface as a capsular polysaccharide (CPS). Rap proteins, extracellular protein substrates of the PrsDE type I secretion system (TISS), share at least one Ra/CHDL (*cadherin-like*) domain and are involved in biofilm matrix development either through cleaving the polysaccharide by Ply glycanases or by altering the bacterial adhesive properties. It was shown that the absence or excess of extracellular RapA2 (a monomeric CPS calcium-binding lectin) alters the biofilm matrix’s properties. Here, we show evidence of the role of a new Rap protein, RapD, which comprises an N-terminal Ra/CHDL domain and a C-terminal region of unknown function. RapD was completely released to the extracellular medium and co-secreted with the other Rap proteins in a PrsDE-dependent manner. Furthermore, high levels of RapD secretion were found in biofilms under conditions that favor EPS production. Interestingly, size exclusion chromatography of the EPS produced by the Δ*rapA2*Δ*rapD* double mutant showed a profile of EPS molecules of smaller sizes than those of the single mutants and the wild type strain, suggesting that both RapA2 and RapD proteins influence EPS processing on the cell surface. Biophysical studies showed that calcium triggers proper folding and multimerization of recombinant RapD. Besides, further conformational changes were observed in the presence of EPS. Enzyme-Linked ImmunoSorbent Assay (ELISA) and Binding Inhibition Assays (BIA) indicated that RapD specifically binds the EPS and that galactose residues would be involved in this interaction. Taken together, these observations indicate that RapD is a biofilm matrix-associated multimeric protein that influences the properties of the EPS, the main structural component of the rhizobial biofilm.

## Introduction

Most bacteria live in complex communities known as biofilms, which confer them several adaptive advantages, like resistance to antimicrobials, oxidative damage and desiccation, among many other beneficial traits (Davey and O’Toole, 2000; Danhorn and Fuqua, 2007). Biofilms are complex bacterial aggregates embedded in an extracellular polymeric substance produced by themselves, other microorganisms, or even eukaryotic hosts (Secor et al., 2018). Over the decades, several pieces of evidence have been gathered on the importance of the extracellular matrix for proper biofilm development, bacterial survival, pathogenicity, and tolerance to the host’s immune response (Danhorn and Fuqua, 2007; Dragos and Kovacs, 2017; Reichhardt and Parsek, 2019). The functional and structural components of the biofilm matrix may include soluble polysaccharides, proteins, eDNA (Flemming and Wingender, 2010), and inorganic components (Keren-Paz and Kolodkin-Gal, 2020). The presence of organic insoluble constituents such as amyloid fibers, cellulose, fimbriae, pilli, and flagella has also been described (Serra et al., 2013; Hobley et al., 2015).

Although many studies have shown the importance of polymers such as the polysaccharides as key structural components of the biofilm matrix, few of them were focused on the role(s) of matrix-associated proteins in either biofilm development, their interaction with other extracellular matrix components, or in the bacteria-host interactions. One of the best examples was reported for *Vibrio cholerae* in which several matrix-associated proteins play complementary architectural roles during biofilm development (Berk et al., 2012). For *Pseudomonas aeruginosa*, the LecB matrix protein has been shown to bind and direct the Pel polysaccharide towards specific regions in the biofilm (Passos, da Silva et al., 2019) whereas Ecotin, another matrix borne protein, modulates the host’s immune response (Tseng et al., 2018). These examples are not only found in Gram-negative bacteria but in Gram positive as well; in *Streptococcus pneumoniae*, the LytB *N*-acetylglucosaminase was proposed to have a structural role in matrix development, in addition to the enzymatic one (Domenech and Garcia, 2020).

*Rhizobium leguminosarum* is a soil, Gram negative, alphaproteobacterium that has the ability to form nitrogen-fixing nodules in peas, beans, and other legumes. It was proposed that a biofilm lifestyle would allow rhizobia to cope with the low nutrient and harsh soil environment and the competitive rhizosphere niche. *R. leguminosarum* has been shown to form structured biofilms *in vitro* in minimal medium (Russo et al., 2006) and bacterial aggregates on roots (Russo et al., 2015). The exposed moiety of the LPS is indispensable for the tight lateral junctions between bacterial cells and the formation of compact microcolonies (Russo et al., 2015). In addition, in *R. leguminosarum* the formation of a structured biofilm requires the production of the acidic and complex polysaccharide (Russo et al., 2006), which consists of a branched octasaccharide repeating unit containing glucose, glucuronic acid and galactose in a 5:2:1 proportion in addition to pyruvyl and *O*-acetyl sugar substitutions (Skorupska et al., 2006). Although this polysaccharide is mostly released into the extracellular medium as an exopolysaccharide (EPS), part of it is retained on the bacterial cell surface as a capsule (CPS). It is believed that some differences in the non-sugar substitutions would define their final localization (Philip-Hollingsworth et al., 1989; O’Neill et al., 1991). The polysaccharide chains that are synthesized and exported outside the bacterium are normally cleaved on the cell surface by two β1,4-glycanases, PlyA and PlyB, both secreted by the PrsDE Type I secretion system (TISS) (Finnie et al., 1998; Zorreguieta et al., 2000) still resulting in very high molecular weight molecules with a characteristic profile (Vozza et al., 2016). Processing of the polysaccharide on the cell surface by the Ply glycanases was required for the generation of a structured biofilm in static cultures (Russo et al., 2006).

The TISS allows proteins to be secreted directly from the cytoplasm to the external medium across both the inner and the outer membranes by a single, energy-coupled process. It consists of an ABC inner membrane component, an outer membrane component called outer membrane protein (OMP) and a protein from the membrane fusion protein (MFP) family anchored to the inner membrane which recruits the OMP to translocate the protein substrates to the external medium (Delepelaire, 2004; Holland et al., 2005). The chromosomal PrsDE system of *R. leguminosarum* is a particular TISS since it mediates the secretion of an unusually high number of substrates whose genes are dispersed over the genome (Finnie et al., 1997; Russo et al., 2006; Krehenbrink and Downie, 2008). These substrates include proteins from the Rap family that exhibit affinity for the rhizobial cell surface and share at least one Ra/cadherin-like (CHDL) domain, which consists of 100–120 amino acids with a high degree of similarity (Ausmees et al., 2001; Abdian et al., 2013). Among them, the PlyA and PlyB glycanases contain one of these domains in their C-terminal region while the RapA proteins (RapA1 and/or RapA2 depending on the species) consist only of two Ra/CHDL (cadherin-like) domains (Ra1 and Ra2). We demonstrated that RapA2, whose pRL100451 gene is harbored on the pRL10 plasmid, binds to both the EPS and the CPS and that calcium modulates its cadherin-like folding and carbohydrate-binding ability (Abdian et al., 2013). It was shown that RapA is involved in biofilm matrix development and cellular cohesion and that these effects could be due, in part, to an increase in CPS production and a variation in the EPS profile (Vozza et al., 2016).

The protein encoded by the chromosomal RL2702 locus (RapD) of *R. leguminosarum* 3841 was also proposed as a member of the Rap family since it contains an N-terminal Ra/CHDL domain, in addition to another C-terminal region of unknown function (Abdian et al., 2013). Therefore, it is possible that the Ra/CHDL domain confers to RapD the ability to bind the acidic polysaccharide and influences some properties of the biofilm matrix as part of the multiple-step process of biofilm maturation. In this work, we show that RapD is co-secreted with the other Rap proteins by the PrsDE TISS. Similar to RapA2, RapD exhibits a calcium-dependent β-structure that is capable of specifically binding the EPS. However, it appears that RapD’s function differs from that proposed for RapA2 since, unlike RapA2, it is not retained on the bacterial surface but completely released to the extracellular medium, while RapA2 is monomeric, RapD tends to form multimers.

## Materials and Methods

### Bacterial Strains and Growth Conditions

*Rhizobium leguminosarum* strains used in this work were *Rhizobium leguminosarum* bv. *viciae (Rlv)* 3841 (Johnston and Beringer, 1975) and derivative mutants *prsD*::Tn*5* (Krehenbrink and Downie, 2008), *pssA*::Tn*5* (Williams et al., 2008) and Δ*rapA2* (Vozza et al., 2016). Strains were grown in TY (Beringer, 1974) or Y-minimal medium (Sherwood, 1970) containing mannitol (0.2 % wt/vol) as the carbon source at 28°C with the appropriate antibiotics. Bacterial growth was monitored at 600 nm using a visible light spectrophotometer. Plasmids were mobilized into *Rhizobium* spp. by triparental mating using *Escherichia coli* RK600 as a helper strain (Finan et al., 1986) or by biparental mating using the *E. coli* S17 strain (Quandt and Hynes, 1993) (for plasmid pK18*mobsacB*). Antibiotics in the culture media were used in the following concentrations: streptomycin 400 μg/ml, kanamycin 50 μg/ml, gentamycin 20 μg/ml, and chloramphenicol 50 μg/ml.

Biofilm-assocciated phenotypes were analyzed in *Rlv* 3841 derivated strains as previously described for polystyrene adhesion (Russo et al., 2006), macrocolony morphology (Vozza et al., 2016) and swimming and swarming motilities (Sorroche et al., 2018).

### Generation of Mutant and Overexpression Derivative Strains

A *rapD* deletion mutant was generated in *R leguminosarum* bv. *viciae* 3841 strain by homologous recombination of the Δ*rapD* carried in the suicide plasmid pK18*mobsacB*/Δ*rapD*. Briefly, 2 sets of primers (Δ*rapD*-A-fwdBamHI: GAGTAGGGATCCT TCTTCTTCTCATAGGCGT, Δ*rapD*-A-revlinker: TCGATACT CTCCTTCTTTCAGTGGCTTTGCATTTGTCATTTG, Δ*rap*D-B-fwdlinker: CTGAAAGAAGGAGAGTATCGATTTTCGCTGT TTGCCTTGCTA and Δ*rap*D-B-revBamHI: ATTGGATCCC AATCCGACCCAGTGCGAG) were used to amplify proximal and distal ends of *rapD* gene (Uniprot: Q1MFT2, locus tag: RL2702) with a random linker sequence. The 1244 bp fragment obtained by recombinant PCR amplification was BamHI-digested and cloned into pK18*mobsacB* in the same restriction site (Schafer et al., 1994). The pK18*mobsacB*/Δ*rapD* plasmid was transferred from *E. coli* S17 to *Rlv* 3841 strain by biparental mating. Single cross-over events were selected on a Y-minimal medium (to avoid *E. coli* growth) supplemented with streptomycin and kanamycin. Then, single colonies were picked and grown overnight in liquid culture without antibiotics. Double recombinants were selected from TY agar plates containing 10% sucrose and streptomycin and kanamycin sensitivity was confirmed. The deletion of *rapD* was verified by PCR using the oligonucleotides Δ*rapD*-A-fwdBamHI and *rapD*full-revXbaI: GCTCCCTCTAGACTATAGCAAGGCAAACA.

The double mutant Δ*rapA2*Δ*rapD* was also generated by homologous recombination but in a Δ*rapA2* mutant strain background.

To overexpress RapD, *rapD* coding sequence (locus tag: RL2702, 834 nt) was amplified from 3841 strain using the oligonucleotides *rapD*full-fwdKpnI: GAAGGTACCGACA AATGCAAAGCCAACT and *rapD*full-revXbaI, which generates a product of 853 pb that was double digested with KpnI and XbaI and cloned in pBBR1-MCS2 (Kovach et al., 1995) under the *lacZ* promoter. Plasmid pBBR1*rapD* was then transferred to *Rlv* 3841 derivative strains via triparental mating using the helper strain *E. coli* RK600. The selection of rhizobial transconjugants was done on TY rich media agar plates using streptomycin and kanamycin (for single mutant or wild type strains) and gentamycin (for double mutant Δ*rapA2*Δ*rapD*). The presence of plasmid pBBR1*rapD* was checked by PCR using T7 and T3 universal primers.

### Generation of a Polyclonal Anti-RapD Antiserum

To generate specific antibodies against RapD, a truncated version of RapD was constructed using the coding sequence of RapD without the Ra/CHDL domain (aa 122 – 277) (Uniprot: Q1MFT2, locus tag: RL2702). This region was amplified from *Rlv* 3841 using the primers *rapD*-NdeI-fwd: GGAGGCCATATGTACCATGAAAACCTGGAT and *rapD*-NotI-rev: CTCGCGGCCGCATATAGCAAGGCAAA. The NdeI-NotI double digested product was cloned into the same restriction sites of plasmid pET-22b to generate the vector pET-22b*rapD*trunc. After induction of the *E. coli* BL21 (DE3) cultures carrying pET-22b*rapD*trunc with 0.5 mM isopropylthiogalactoside overnight at 18°C (OD of 0.6–0.8), cells were resuspended in a buffer containing 20 mM Tris-HCl, 0.5 M NaCl, pH 8 with the addition of 1 mM phenylmethanesulfonyl fluoride and 10 mM imidazole. Cells were disrupted in a French pressure cell at 18,000 p.s.i. Then, the cell extract was centrifuged at 100,000 × *g* for 1 h and the supernatant was applied to a Ni-NTA HiTrap Chelating column (GE Healthcare, Amersham, United Kingdom) (#RPN2232) and eluted with a buffer containing 500 mM imidazole during a lineal gradient. Fractions containing truncated RapD were pooled and concentrated by ultrafiltration. Mouse polyclonal antisera against the truncated form of RapD was generated by injecting the SDS-PAGE 15% gel-excised band of 18 kDa containing the fragmented RapD into BALB/c mice. The initial dose started with 100 μg of protein/mouse using Freund’s complete adjuvant. Each mouse received the same concentration of booster every 2 weeks with Freund’s incomplete adjuvant up to a total of four immunizations.

The polyclonal antisera against RapD were performed using the animal procedures and management protocols approved by the local Institutional Committee (“Comité de Bioética Fundación Instituto Leloir”) protocol number 2009 01 BFIL.

### Purification of Recombinant Full-Length RapD

Complete RapD coding sequence (aa 1 – 277) (Uniprot: Q1MFT2, locus tag: RL2702) was amplified using primers *rapD*full-NdeI-fwd: GCCCTCCATATGACAAATGCAAAGCCA ACT and *rapD*-NotI-rev: CTCGCGGCCGCATATAGCAAGGCA AA and the NdeI-NotI-digested product of 854 pb was cloned in pET-22b digested with the same restriction endonucleases to obtain pET22b*rapD*. *E. coli* BL21 (DE3) carrying pET22b*rapD* (OD of 0.6–0.8) was incubated with 0.1 mM isopropylthiogalactoside overnight at 18°C and cells were resuspended in a buffer containing 20 mM Tris-HCl, 0.5 M NaCl, pH 8 with the addition of 10 mM imidazole and 1 mM CaCl_2_. Then, cells were disrupted in a French pressure cell at 18,000 p.s.i. The cell extract was centrifuged at 100,000 × *g* for 1 h, the supernatant was applied to a Ni-NTA HiTrap Chelating column (GE Healthcare, Amersham, United Kingdom) (#RPN2232) and eluted with a buffer containing 500 mM imidazole during a lineal gradient. Fractions containing full-length RapD were dialyzed against 20 mM Tris-HCl, 0.15 M NaCl, 10 mM EGTA, pH 8 overnight and applied to Superdex 200 column (Pharmacia Corp.) pre-equilibrated in 20 mM Tris-HCl and 150 mM NaCl, pH 8. Purified full-length RapD was pooled, concentrated by ultrafiltration, and stored at –80°C.

### Analysis of Extracellular and Surface Proteins

To analyze secreted proteins, rhizobia were grown at 28°C and monitored at 600 nm with a visible light spectrophotometer. Culture supernatant proteins were concentrated by precipitation with 10% trichloroacetic acid (TCA) as described previously (Economou et al., 1990). After precipitation, TCA was removed by washing the precipitate with cold acetone twice. For SDS-PAGE analysis and Western blot, TCA precipitates were resuspended with a loading buffer containing urea and β-mercaptoethanol.

For LC-MS/MS analysis, starter cultures were diluted to an OD of 0.01 and grown for approximately 24 h in TY rich media to an OD of 0.7–0.8 (exponential growth phase). TCA precipitates were resuspended with 50 mM ammonium bicarbonate, quantified using Bio-Rad Bradford reagent according to manufacturer instructions, and analyzed by the CEQUIBIEM service (Departamento de Química Biológica, Facultad de Ciencias Exactas y Naturales, UBA). Cytoplasmic protein RhiA (Krehenbrink and Downie, 2008) was used as a lysis marker for the extracellular samples.

Analyses of surface proteins were performed with equivalent amounts of cells as follows. Starter cultures were diluted to an OD of 0.01 and grown for 24 h in rich (TY) or minimal (Y) medium to an OD of 0.6–0.8 (exponential growth phase). Standardized bacterial pellets were washed with Tris 30 mM pH 8.4 and resuspended in a high ionic strength buffer containing 30 mM Tris pH 8.4 and 1.5 M NaCl. Cells were submitted to mechanical pressure using a 27-gauge needle 20 times. Bacterial debris was removed, the supernatant was centrifuged for 1 h at 21,000 × *g* and the proteins were precipitated with TCA 10% as described above.

### Western Blot

After SDS-PAGE, proteins were transferred to a PVDF membrane (Millipore, Billerica MA, United States), blocked with 5% skimmed milk in TBS-0.05% Tween 20 and incubated with the proper antibodies: Primary antibodies against RapA2, diluted 1:5000 (Abdian et al., 2013) or RapD, diluted 1:8000 were prepared in TBS-0.05% Tween with 1% BSA. RapA2 was revealed by an anti-rabbit secondary antibody conjugated to HRP and RapD antiserum was revealed by an anti-mouse secondary antibody conjugated with HRP (Jackson Immunoresearch Laboratories Inc., United States) (#115-036-062). Secondary antibodies conjugated with HRP were revealed with ECL (GE Healthcare, Amersham, United Kingdom) (#RPN2232) according to manufacturer instructions.

To compare RapD secretion under different culture conditions, the coefficient between the specific RapD signal and total proteins signal was estimated. Coomassie blue staining of total proteins was analyzed by densitometry with the ImageJ software (NIH, United States) (https://imagej.nih.gov/ij/download.html). For RapD-specific signals, Western blots were also quantified by densitometry using the same software.

### Colony Blots

For colony blots, 10 μl of standardized bacterial suspensions from 48 h starter cultures (OD 0.05) were grown at 28°C on a sterilized nitrocellulose filter placed on TY or Y agar plates. After 72 h, the macrocolonies were washed away from the nitrocellulose filter with MilliQ, and immunodetection was performed as described above for the Western blot analysis.

### Immunofluorescence

To analyze RapD localization, immunofluorescence analyses of whole bacterial cells were performed. Starter cultures were diluted to an OD of 0.025 and grown for 24 h in rich TY medium with proper antibiotics. Cells were harvested during exponential growth (OD 0.8) and fixed with paraformaldehyde 4% in PBS at 28°C for 20 min. After blocking with gelatin 0.2% for 1 h at room temperature, incubation with an anti-RapD antiserum (1:200) in PBS-BSA 1% for 1.5 h at room temperature was performed. Then, anti-mouse secondary antibodies conjugated with Cy3 (1:200) in PBS-BSA 1% were incubated for 1 h at room temperature. Cells were spotted onto glass slides with agarose 1% pads and visualized in a Zeiss Axio Observer 3 inverted fluorescence microscope (Carl Zeiss Microscopy, LLC, United States). For images, the fluorophore signal was pseudo-colored cyan.

### Preparation and Analyses of the Exopolysaccharide

The extracellular medium was obtained from culture supernatants as follows. Cells were grown in Y- mannitol medium for 5 days at 28°C. NaCl was added to a final concentration of 0.25 mM in cell-free supernatants and EPS was precipitated with 2½ volumes of 100% ethanol. EPS precipitate was washed with increasing ethanol concentrations from 70 to 90% and let to air dry before being resuspended in MilliQ water. Polysaccharides were quantified by the determination of hexuronic acids by the meta-hydroxidiphenyl method (Filisetti-Cozzi and Carpita, 1991). Proteinase K treated polysaccharide was obtained by incubating 1 mg of uronic acids equivalents with 2 mg/ml proteinase K at 60°C for 1 h. Proteinase K was heat-inactivated at 28°C for 40 min and centrifuged at 16,000 × *g* for 30 min. Soluble EPS was then precipitated as described above.

### Size Exclusion Chromatography

Extracellular medium preparations (1 mg/ml uronic equivalents) were heated at 100°C for 10 min and centrifuged at 10,000 × *g* to remove denatured proteins as described previously (Vozza et al., 2016). Treated EPS was fractioned by gel filtration chromatography in a Superose 6 HR 10/30 column (Amersham Biosciences) previously equilibrated in 0.1 M NaCl and 0.1 M sodium phosphate buffer pH 7. Hexoses in every 1 ml fraction were quantified according to Loewus (1952).

### Enzyme-Linked ImmunoSorbent Assay Direct Binding and Binding Inhibition Assay

The interaction between RapD and the EPS from the *Rhizobium leguminosarum* 3841 strain was analyzed as described previously (Abdian et al., 2013) with some modifications. Briefly, the microplates were coated with 100 μl of a 0.1 mg/ml EPS/Xanthan solution in binding buffer (50 mM sodium carbonate pH 9.7) overnight at 4°C. For conventional Enzyme-Linked ImmunoSorbent Assay (ELISA), increasing concentrations of RapD ranging from 0.05 μg/ml to 10 μg/ml were incubated in TBS, 1 mM CaCl_2_ for 2 h at room temperature. For binding inhibition assays (BIA), RapD (1 μg/ml) was preincubated before addition to the wells with serial 10-fold dilutions of the putative inhibitory compound for 30 min at room temperature. The initial concentration of the inhibiting polysaccharides was 0.1 mg/ml and 5 mM for each monosaccharide. Bound RapD was detected with a polyclonal antiserum against the C-terminal domain of RapD (truncated RapD) and revealed with an HRP conjugated secondary antibody. OPD was used as a substrate in the peroxidase reaction which was stopped after 4 min and absorbance at 492 nm was measured with a multiwell scanner. The data represent the mean values of a representative experiment of three independent assays done in triplicate.

### Protein–Protein Binding Inhibition

The effect of RapA2 on RapD-EPS interaction was carried out using a Binding-Inhibition Assay (BIA). Fixed EPS was either preincubated with increasing RapA2 concentrations (0.05 μg/ml to 10 μg/ml) for 2 h at room temperature or incubated simultaneously with RapA2 (0.05 μg/ml to 10 μg/ml) and RapD (5 μg/ml) for 2 h at room temperature. Then, proteins were washed and the remaining bound RapD was quantified following the procedure mentioned above.

### Far Ultraviolet Circular Dichroism Spectroscopy

Analyses were done in 15 mM Tris pH 8, 15 mM NaCl with the addition of the corresponding CaCl_2_ (0–1.25 mM), NaCl (0–500 mM) or EPS concentration (0–12 μg/ml uronic equivalents). Far-Ultraviolet Circular Dichroism (Far-UV-CD) measurements were carried out at 25°C on a Jasco J-815 spectropolarimeter (JASCO Corp, Japan). The recombinant RapD protein was diluted to 3.75 μM (0.12 mg/ml) and placed in a cell with a 1-mm path length. Spectra were acquired over the wavelength range of 195–260 nm. Measurements were performed in a Peltier-thermostatted cell holder using a 1-mm path length cell. The spectra acquired were the average of six scans to reduce background noise.

### Fluorescence Spectroscopy

Fluorescence emission spectra of recombinant RapD were recorded at 25°C between 305 and 400 nm with an excitation wavelength of 295 nm using excitation and emission bandwidths of 5 nm in a Jasco FP-6500 spectrofluorometer (JASCO Corp., Japan). Fluorescence emission data were analyzed by first subtracting the buffer background. The RapD protein concentration was 3.75 μM.

### Oligomerization Analysis

A total of 100 μl of RapD (1 mg/ml) were loaded on a Superdex 200 column (Pharmacia Biotech, Cytiva, United States) (#17-5175-01) and size exclusion chromatography was carried out at 1 ml/min in Tris 20 mM pH 8, 150 mM NaCl and CaCl_2_ (ranging from 0.1 to 1.25 mM). Fractions (1 ml) were concentrated by precipitation with 10% TCA, resuspended in loading buffer, and analyzed by SDS-PAGE. Molecular weight markers used were Ferritin (440 kDa), BSA (67 kDa), OVA (45 kDa), and Lysozyme (15 kDa). Blue dextran and acetone were used for V0 and V0+Vi calibration.

### Static Light Scattering Measurements

The average MW of RapD in solution was determined on a Precision Detectors PD2010 90° light scattering instrument (Bellingham, MA, United States) tandemly connected to high-performance liquid chromatography and an LKB 2142 differential refractometer. A Superdex 200 column was used. Hundred μl of the purified recombinant RapD at 1 mg/ml pre-incubated with 1.25 mM CaCl_2_ was injected into the column, and the chromatographic runs were performed with a buffer containing 20 mM Tris-HCl, 0.15 M sodium chloride, pH 8, under isocratic conditions at a flow rate of 0.4 ml min^–1^ at 20°C. The molecular weight of each sample was calculated relating its 90° and RI signals and comparison of this value with the one obtained for BSA (molecular mass: 66.5 kDa) as a standard using the software Discovery32. The average and standard deviation values correspond to the peak center ± 0.08 ml.

### Comparative Analyses for RapD Protein

The coding sequence for RapD (RL2702) (Supplementary Table 1) was used as a query for a protein blast analysis excluding *R. leguminosarum* species and limited to *Rhizobium* genus. The orthologous proteins found were used in local pairwise alignments to detect similarities as well as for global alignment analyses with Clustal Omega (EMBL-EBI, United Kingdom).

## Results

### RapD Is Co-secreted With the Other Rap Proteins by the PrsDE TISS System

**FIGURE 1:**
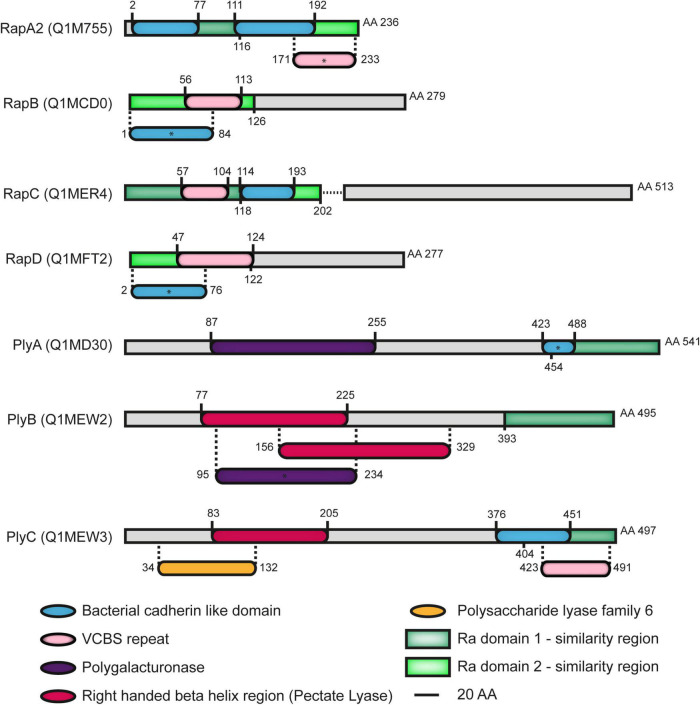
Domains of Rap family of proteins secreted by the PrsDE system in *Rhizobium leguminosarum* bv. v*iciae* (*Rlv*) strain 3841. Ra1 and Ra2 Ra/CHDL domains from RapA2 are represented with dark and light green boxes respectively. Regions that align with those domains are depicted as the corresponding green boxes in other Raps. RapC is truncated in *Rlv* 3841 (aa 202), in contrast with other strains analyzed, due to a chromosomal deletion of five nucleotides which generates a stop codon shown in the scheme as a dotted line. (*) Asterisks indicate the presence of domains evidenced after setting the *E*-value threshold to 0.1 with the exception of RapB in which the *E*-value threshold was set to 0.2 (default 0.01). Ra/CHDL domains consist of about 110 residues found in several bacterial proteins with a high degree of structural similarity with the cadherin-family of eukaryotic calcium-binding proteins involved in cell-to-cell interactions. Ra domains (Ra1 and Ra2) were defined as bacterial CHDL domains (Ausmees et al., 2001; Abdian et al., 2013). CHDL domains are present in numerous bacterial and archaeal proteins and were previously proposed to confer carbohydrate-binding ability (Cao et al., 2005). Polygalacturonase, pectate lyase, and polysaccharide lyase family 6 correspond to domains found in glycanases and are related to their enzymatic activity. VCBS repeats are domains of about 100 residues found in multiple copies in large proteins of *Vibrio*, *Colwellia*, *Bradyrhizobium*, and *Shewanella* whose role is related to cell-to-cell interactions (InterPro domain IPR010221).

In addition to the Ply glycanases, the RapA lectins and the RapB and RapC proteins, another member of the Rap family, named RapD (RL2702), was predicted by an *in silico* search of Ra/CHDL domain-harboring proteins in the *Rlv* 3841 genome (Abdian et al., 2013). The Ra/CHDL domains consist of about 110 residues found in several bacterial proteins with a high degree of structural similarity with the cadherin-family of eukaryotic calcium-binding proteins involved in cell-to-cell interactions. CHDL domains are present in numerous bacterial and archaeal proteins and were previously proposed to confer carbohydrate-binding ability (Cao et al., 2005). RapA1 and RapA2 lectins are the smallest members of the Rap family containing only two Ra domains (Ra1 and Ra2) (Ausmees et al., 2001; Abdian et al., 2013). Both the size and the domain structure of RapD are similar to those of RapB since it contains an N-terminal Ra2 domain (aa. 1–122) that includes a VCBS repeat (aa. 47–124) and a C-terminal region (aa. 123–277) of unknown function, sharing an overall 49% similarity with RapB (aa. 1–248) (Figure 1; Supplementary Table 1). The VCBS repeats comprise about 100 residues and are domains found in multiple copies in large proteins of *Vibrio*, *Colwellia*, *Bradyrhizobium*, and *Shewanella* whose functions are related to cell-to-cell interactions. Local (PBlast) and global (Clustal) alignments showed a degree of similarity greater than 85% between the RapD orthologues of species belonging to the *Rhizobium* genus such as *R. indigoferae, R. laguerrae, R. ecuadorense, R. valis, R. phaseoli, R. etli, and R. esperanzae*, among others (Supplementary Figure 1), suggesting that RapD is highly conserved in this genus. Iterative BLAST using as query sequences either the complete or the C-terminal region resulted in no orthologous outside the *Rhizobium* genus.

In order to determine if RapD is a substrate of the PrsDE system and is co-secreted with the other PrsDE-dependent proteins, the secretome of the *Rlv* 3841 wild type strain and the isogenic *prsD*::Tn*5* secretion mutant was analyzed by LC/MS-MS as described in Methods. Several proteins were absent in the extracellular medium of the *prsD* mutant. As was previously shown (Russo et al., 2006; Krehenbrink and Downie, 2008), the secretion of RapA2 (Q1M755), RapC (Q1MER4), PlyA, and PlyB (Q1MD30 and Q1MEW2) was PrsDE-dependent (Table 1). In addition, the RapD protein (RL2702) was found in the secretome of the wild type strain but was absent in the extracellular medium of the *prsD* secretion mutant. The secretion of other PrsDE-dependent proteins such as three calcium-binding RTX proteins (Q1MF23, Q1M7J7, and Q1MGL7), a beta-helix domain-containing protein (Q1M8U8), and an NDK-nucleoside diphosphate kinase (Q1MIY5) was corroborated (Krehenbrink and Downie, 2008). Besides, three novel PrsDE dependent extracellular proteins were detected in *Rlv* 3841: a calcium-binding RTX Zn-metalloprotease (Q1ML69), the RapB protein (Q1MCD0), and the PlyC glycanase (Q1MEW3). These proteins were previously proposed *in silico* as putative TISS substrates (Krehenbrink and Downie, 2008; Table 1 and Supplementary Table 1).

**TABLE 1:** PrsDE-dependent extracellular proteins of *R. leguminosarum* bv. *viciae* 3841.

Locus Tag/Code	Predicted MW (kDa)	Description	References
RL3024/Q1MEW2	51.6	PlyB	Finnie et al., 1998
RL0790*/Q1ML69	160	Putative calcium binding RTX Zn-metalloprotease	Krehenbrink and Downie, 2008
pRL90140/Q1M8U8	78.9	Beta helix domain-containing protein	Krehenbrink and Downie, 2008
RL2961/Q1MF23	95.1	Putative calcium-binding cadherin-like RTX protein	Krehenbrink and Downie, 2008
pRL100175/Q1M7X8	30	NodO	Downie and Surin, 1990; Finnie et al., 1997)
pRL100309/Q1M7J7	60.8	Putative calcium-binding cadherin-like RTX protein	Krehenbrink and Downie, 2008
pRL100451/Q1M755	24.9	RapA2	Ausmees et al., 2001; Russo et al., 2006
RL3659*/Q1MD30	56.1	PlyA	Finnie et al., 1998
RL2412/Q1MGL7	64.6	Putative calcium binding RTX protein	Krehenbrink and Downie, 2008
RL2702*/Q1MFT2	30.6	RapD	Abdian et al., 2013
RL3023*/Q1MEW3	51.8	PlyC	Krehenbrink and Downie, 2008
RL3073/Q1MER4	30.3	RapC (C terminus ORF)	Ausmees et al., 2001; Russo et al., 2006
RL3911*/Q1MCD0	30.3	RapB	Ausmees et al., 2001
RL1580/Q1MIY5	15.3	NDK – nucleoside diphosphate kinase	Chakrabarty, 1998; Krehenbrink and Downie, 2008

*Asterisk indicates novel PrsDE substrates. Rap proteins are ordered from highest to lowest score (indicative of abundance and number of tryptic peptides found).*

**FIGURE 2:**
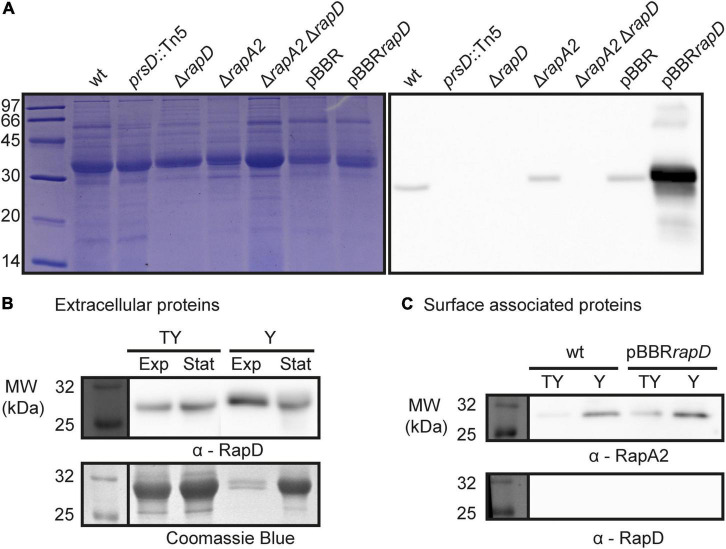
RapD secretion: PrsDE dependence and final destination. **(A)** Extracellular proteins of spent cultures from several *Rlv.* 3841 isogenic strains were analyzed by 15% SDS-PAGE and Western blot. Immunodetection of secreted RapD was revealed by specific anti-RapD antiserum revealed by an anti-mouse HRP conjugated secondary antibody. **(B)** RapD secretion in nutrient-rich (TY) and deprived (Y) conditions. Western blot analysis of extracellular proteins from exponential phase [OD 600 nm = 0.7 (TY) and 0.8 (Y)] and late stationary phase [OD 600 nm = 1.2 (TY) and 1.5 (Y) cultures are shown]. **(C)** Surface-associated proteins from exponential phase cultures of *Rlv* 3841 wild type strain and a RapD overexpressing strain cultured in rich (TY) and minimal (Y) medium. Western blot was revealed using polyclonal antisera against RapA2 or RapD and a HRP conjugated secondary antibody.

The presence of RapD in the extracellular medium of *Rlv* 3841 grown in TY rich medium was confirmed by Western blot using specific polyclonal antibodies generated against the C-terminal region of RapD (Figure 2). We also included in this analysis a mutant that was obtained by deletion of the *rapD* gene (Δ*rapD*) and a wild type derivative-strain that overexpresses *rapD* from a multicopy plasmid (pBBR*rapD*). In line with the proteomic analysis, RapD was absent in the extracellular medium of the *prsD* secretion mutant (Figure 2A). High levels of extracellular RapD were observed in the *rapD*-overexpressing cells from pBBR*rapD*. In relation to the total extracellular protein amount produced by the wild type strain both in rich (TY) and Y-mannitol minimal media, RapD showed a steady accumulation. However, an estimation by densitometry using ImageJ suggests that the amount of RapD in the extracellular medium at an OD of 0.8 in minimal Y-mannitol medium in relation to the total extracellular proteins was considerably higher (up to fourfold) than that observed in the rich medium (Figure 2B). This observation is interesting since copious amounts of EPS are produced in Y-mannitol and *Rlv* forms structured biofilms in this medium both *in vitro* and on pea roots (Russo et al., 2006; Williams et al., 2008; Vozza et al., 2016). Taken together, these results show that RapD is co-secreted with the other Rap(s) in a PrsDE-dependent manner. Noteworthy, RapD was detected in the wt strain either as a faint or a strong band that slightly differs in the molecular weight between TY or Y media (Figure 2B). The reasons for the difference in RapD behavior under different culture conditions are so far unknown.

**FIGURE 3:**
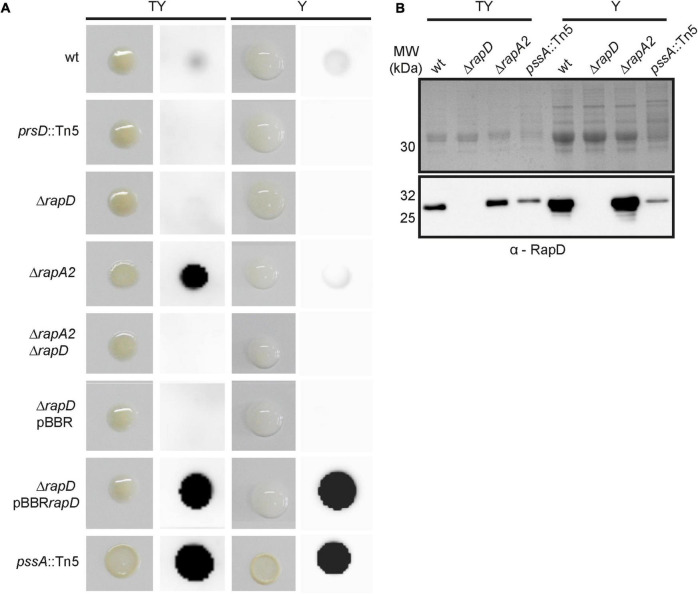
RapD colony blot. **(A)** The macroscopical aspect of the macrocolonies of several *Rlv* 3841 derivative strains grown in TY and Y-minimal media at 28°C and Immuno-detection of secreted RapD retained on the nitrocellulose membrane placed on semisolid medium using specific anti-RapD antiserum. **(B)** Western blot analysis of extracellular proteins secreted by *Rlv* 3841 derivative strains after 72 h analyzed by 15% SDS-PAGE. Immunodetection of secreted RapD was revealed by an anti-mouse secondary antibody HRP-conjugated.

To determine whether RapD is also secreted in biofilms, macrocolonies that are developed on semisolid media were used as a biofilm model (Branda et al., 2005; Tseng et al., 2018). A moderate signal corresponding to extracellular RapD was detected in the wild type strain and, as expected, absent in the *prsD* and Δ*rapD* mutants, both in TY and Y media (Figure 3A). Besides, a very strong signal was observed in the membrane where the macrocolony of the *rapD*-overexpressing strain (pBBR*rapD*) was grown. These observations support the idea that RapD is secreted under sessile conditions. In order to analyze RapD secretion in the macrocolony of a strain deficient in EPS production, the *pssA* mutant (Williams et al., 2008) was also analyzed. Surprisingly, for this strain both in TY and Y media, a strong extracellular RapD signal was observed (Figure 3A). This seems not to be due to an increased expression/secretion of RapD in the EPS-deficient strain since analysis by Western blot of the extracellular proteins showed RapD levels that were even lower than those of the wild type strain (Figure 3B). As expected, colonies of the *pssA* mutant were visibly dry on both TY medium and Y medium (Figure 3A). Therefore, the strong signal of extracellular RapD may be related somehow to the EPS-deficient phenotype.

RapA lectins are partly retained by the capsular polysaccharide (CPS) on the cell surface and modulate cellular cohesion (Vozza et al., 2016). In order to evaluate the interplay between RapA and RapD, a RapA2-deficient strain was cultured under the same sessile conditions. Similar to that observed for the EPS-defective mutant, a prominent RapD signal was observed in the Δ*rapA2* mutant grown in TY medium, in which this strain shows a dry colony phenotype (Vozza et al., 2016; Figure 3A). Again, the levels of extracellular RapD in this strain grown under planktonic conditions in the TY medium were similar to that of the wild type (Figure 3B), suggesting that the high RapD signal is not due to an increased RapD secretion. Since the *pssA* mutant (on both Y-mannitol and TY media) and the *rapA2* mutant (only on TY) develop dry macrocolonies (Figure 3A; Vozza et al., 2016), the simplest explanation for these observations is that extracellular RapD is more efficiently retained by the nitrocellulose membrane in EPS altered/deficient (dry) colonies while in the mucoid ones a considerable proportion of it is washed out together with the bacterial biomass, probably due to a strong interaction of RapD with the EPS or the CPS. However, another non-exclusive possibility is that RapD secretion in these deficient/altered EPS strains is increased when the bacteria are grown in biofilms.

### RapD Is Completely Released to the Extracellular Medium

**FIGURE 4:**
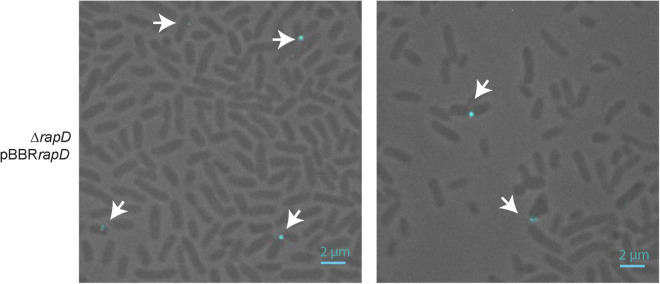
Immunofluorescence of RapD. Representative fields for the RapD overexpressing strain grown for 20 h in TY medium detected with anti-RapD antisera and anti-mouse Cy3–conjugated secondary antibodies are shown. Cells were observed at 1000X. The size bar represents 2 μm and white arrows indicate polar signals of RapD.

As mentioned, upon secretion, a proportion of the RapA protein is retained by the CPS on the bacterial cell surface (Vozza et al., 2016). To explore RapD destination, a surface-associated protein preparation from the wild type strain was analyzed by Western blot using anti-RapD antibodies. In contrast with RapA2, the RapD protein was not detected in the surface-associated protein fraction neither in the rich medium nor in the Y-minimal medium (Figure 2C). Furthermore, RapD was absent in the cell surface fraction of the *rapD*-overexpressing bacteria (Figure 2C). Accordingly, immunofluorescence analysis of the *Rlv* 3841 pBBR*rapD* cells indicated that RapD is almost not retained on the cell surface (Figure 4). Few bacterial cells showed a polar extracellular signal, probably due to a transient detection of the protein after secretion (Supplementary Figure 2). These observations suggest that similar to RapA, RapD is secreted by the PrsDE system in a polar manner.

In conclusion, it seems that the final fate of RapD is different from that of RapA proteins. While RapA proteins are partly retained on the cell surface interacting with the CPS, RapD is completely released into the extracellular environment, probably interacting with the polysaccharide in its released form (EPS).

### Calcium Induces Conformational Changes and the Formation of Oligomers in RapD

**FIGURE 5:**
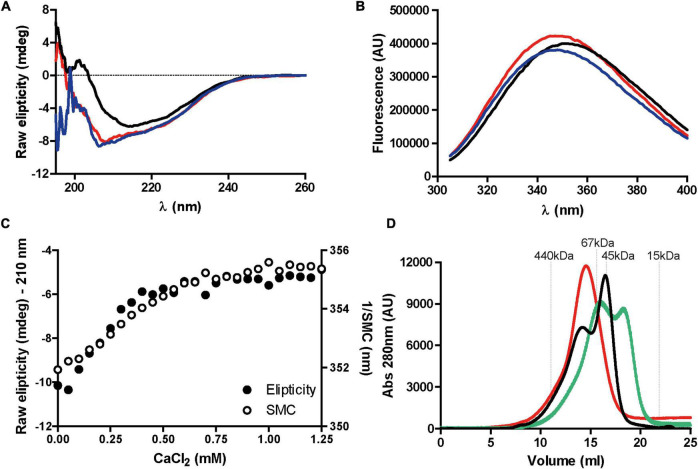
Effect of calcium on RapD structure. **(A)** Circular dichroism spectra of purified recombinant decalcified RapD (Apo-RapD) (3.75 μM) in the absence of calcium (red line), 1.25 mM calcium (black line), or 1.25 mM calcium supplemented with EGTA 3 mM (blue line). **(B)** Fluorescence spectroscopy of RapD (3.75 μM) in response to calcium. Apo-RapD fluorescence in the absence of calcium (red line), with 1.25 mM calcium (black line) and with 1.25 mM calcium supplemented with 3 mM EGTA (blue line) is indicated. **(C)** Raw ellipticity measured at 210 nm and spectral mass center (SMC) of Apo-RapD in response to increasing calcium concentrations (0–1.25 mM) showing simultaneity for secondary structure changes and marginal exposure of the single tryptophan. **(D)** Hydrodynamic changes of RapD (1 mg/ml) in response to calcium. Size exclusion chromatography of recombinant RapD in absence of calcium (red line), 0.75 mM CaCl_2_ (green line) or, 1.25 mM CaCl_2_ (black line). Molecular weight markers used for calibration are indicated.

RapA2 and the N-terminal region of RapD share domain homology with eukaryotic cadherins (Figure 1; Abdian et al., 2013). As calcium ions affect the conformation and dynamics of both eukaryotic cadherins and RapA2, we sought to determine if calcium ions also affect the conformation of RapD protein using circular dichroism (CD) and fluorescence spectroscopy (Figure 5). The CD spectrum of the Apo-RapD (decalcified) is indicative of a protein with a mixed content of secondary structure elements with a minimum centered at ∼210 nm. The addition of calcium in the millimolar range induces a decrease in the ∼210 negative component and the consolidation of a negative band at ∼217 nm, indicating that β sheets dominate the structure of the Ca^+2^-bound RapD (Figure 5A). The change of the CD signal at ∼210 nm saturates with the addition of 0.5 mM of calcium to 3.75 μM of RapD (Figure 5C). The addition of calcium to Apo-RapA2 (Abdian et al., 2013) and to eukaryotic cadherins (Pokutta et al., 1994) also induces the consolidation of protein conformation enriched in β sheets in agreement with the requirement of calcium for the proper folding of RapD cadherin-like domains. On the other hand, the addition of divalent magnesium at the concentrations used in the calcium experiment (Supplementary Figure 3) did not induce any change in the CD spectrum of the Apo-RapD confirming the exquisite preference for calcium ions.

To further investigate the effect of calcium ions on RapD conformation, the Apo-RapD protein was titrated with an increasing concentration of calcium ions following the fluorescent emission spectrum of the unique tryptophan residue (Figure 5B). As shown in Figure 5B, the addition of calcium led to a small red shift in the fluorescent spectrum suggesting that the unique tryptophan residue of RapD present in the Ra/CHDL domain exposes partially to the solvent. The titration curves of RapD with calcium analyzing the CD signal and fluorescence emission can be superimposed indicating that both signals report the same conformational transitions (Figure 5C). Together, these data suggest that calcium-binding induces the stabilization of the β-sheet prone regions and a local rearrangement of the environment of the tryptophan residue that partially exposes itself to the solvent. The addition of 3 mM EGTA to a calcium saturated Rap sample completely restores the CD and the fluorescence emission spectra to that observed for the Apo form indicating that the calcium-induced conformational transition is fully reversible.

It was proposed that cadherins mediate cell to cell contact through dimerization of their extracellular domains and that this process is promoted and maintained by calcium ions (Nagar et al., 1996; Pertz et al., 1999). To evaluate the propensity of RapD to oligomerize in the presence of increasing calcium concentrations we performed size exclusion chromatography (SEC) experiments (Figure 5D). The experiment was performed by loading 100 μl of a 37.5 μM protein sample in a Superdex 200 column. It must be considered that the elution volume observed in a SEC experiment depends on the hydrodynamic radius of the molecule that is, in turn, affected by both the oligomeric state and the conformational compaction. The RapD-Apo protein elutes as a single broad peak at 14.5 ml corresponding to a 118 kDa globular protein. A protein sample pre-incubated with 0.75 mM calcium elutes as two peaks, at 16.1 ml and 18.3 ml respectively. Both peaks correspond to RapD species with a smaller hydrodynamic radius compared to the Apo form. The peak eluting at 18.3 ml behaves as a globular protein of 34.7 kDa and is compatible with the globular monomer form of the RapD protein (MW of 32 kDa). As both protein and calcium initially present in the sample volume dilute upon injection on the column, we analyzed a sample containing 1.25 mM of calcium ion. In this condition, the Rap protein elutes in two peaks at 13.8 and 16.5 ml. These peaks that correspond to protein species with a hydrodynamic radius higher than that of the RapD globular monomer must correspond to oligomeric forms of the compacted monomer, indicating that calcium induces the oligomerization of RapD. Multimerization was verified by an SLS (*static light scattering*) assay in the presence of 1.25 mM CaCl_2_. Two defined peaks of 90 and 32 kDa (A and C) were identified in the presence of 1.25 mM CaCl_2_ (Supplementary Figure 4). Both calculated MWs fall in line with the predicted sizes of the monomer (31.8 kDa) and the trimer (95.4 kDa). The third peak (B) shows a shoulder with an estimated MW of 43 kDa which could be attributed to the equilibrium between the monomeric and dimeric states of 32 and 64 kDa respectively (Supplementary Figure 4). Taken together, SEC and CD titration experiments suggest that calcium induces RapD compaction and a subsequent oligomerization at the expense of the stabilization of the β-sheet secondary structure.

### The Exopolysaccharide Induces Structural Changes in RapD

**FIGURE 6:**
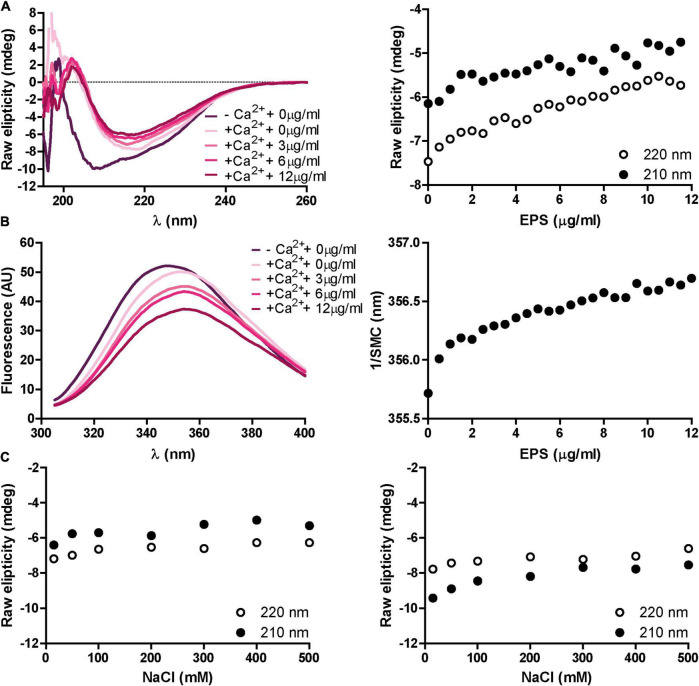
Effect of proteinase K treated EPS on RapD secondary structure and protein environment. **(A)** Representative far-UV CD spectra for decalcified RapD. RapD was incubated in the presence of 1.25 mM CaCl_2_ and 1.25 mM CaCl_2_ with increasing concentrations of EPS and raw ellipticity was measured at 210 and 220 nm. EPS concentrations, ranging from 0 to 0.012 μg/ml are indicated. **(B)** Representative fluorescence spectroscopy spectra for RapD in the absence of calcium, 1.25 mM CaCl_2,_ and 1.25 mM CaCl_2_ with increasing EPS concentrations and spectral mass center in response to EPS. **(C)** Circular dichroism dissociation spectra at increasing NaCl concentrations. Shown in both panels is raw ellipticity at 210 and 220 nm after 30 min of incubation at 25°C for either RapD and EPS in the presence of calcium (left) or RapD and EPS in the absence of calcium (right).

We sought to investigate whether the presence of the EPS exerts an effect on RapD secondary structure. Analysis by CD showed that in the presence of 1.25 mM CaCl_2_, increasing concentrations of the EPS induce a slight change in the secondary structure of RapD, mainly an increase in the ellipticity, which might suggest that the EPS favors the formation of RapD aggregates or multimerization (Figure 6A). The same effect was also observed by fluorescence spectroscopy which showed a spectrum red shift upon the addition of EPS in the presence of calcium. To note, these changes were only seen in the presence of calcium as EPS alone was not able to trigger any change in the protein structure whatsoever (Figure 6B). To discern if non-specific carbohydrate-binding was responsible for the changes observed, RapD was incubated with xanthan, a negatively charged exopolysaccharide produced by *Xanthomonas campestris.* However, xanthan did not trigger the EPS-related conformational changes observed in the previous section (Supplementary Figure 5). The dependence of RapD-EPS interaction to promote these changes was analyzed by CD spectra. RapD was incubated with EPS in the presence or the absence of calcium and increasing concentrations of NaCl (Figure 6C). The results showed that NaCl had no effect on the CD spectra, suggesting that the interaction of RapD-EPS cannot be dissociated by ionic displacement or that once triggered, the conformational changes induced by the EPS cannot be reverted by separating RapD from the EPS.

### RapD Interaction With the EPS

**FIGURE 7:**
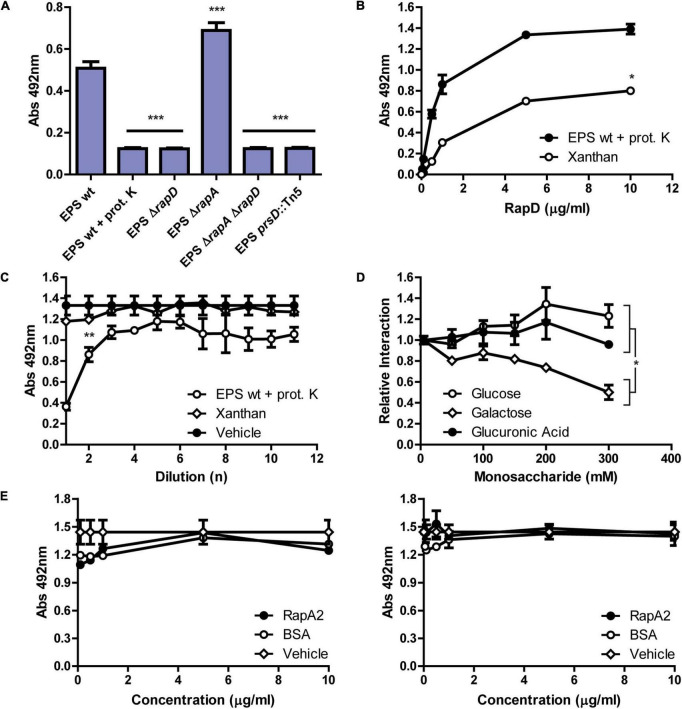
Characterization of the interaction between RapD and the EPS of *R. leguminosarum*. **(A)** Immunodetection of the RapD-EPS interaction by ELISA assay. Crude EPS preparations (0.1 mg/ml) from 5-days-cultures grown in minimal media of several *Rlv* 3841 derived strains were analyzed. The EPSs were immobilized in an ELISA plate and endogenous RapD interaction was revealed using the mouse anti-RapD polyclonal antiserum and an anti-mouse HRP conjugated secondary antibody (Jackson). A representative experiment of 2 independent replicas measured in triplicate is shown. Asterisks indicate statistical differences with the wild type EPS analyzed by one-way ANOVA with an α of 0.05; *p* < 0.0001. **(B)** ELISA assay. Kinetics of the binding of RapD to immobilized proteinase-treated-EPS or Xanthan in the presence of 1 mM CaCl_2_, and revealed using anti-RapD antiserum. 50% binding for RapD-EPS was achieved at 0.5 μg/ml and at 1 μg/ml for RapD-Xanthan. Shown is a representative experiment of three independent experiments measured in triplicate each. An asterisk indicates statistical differences between both polysaccharides analyzed by paired *t*-test with an α of 0.05; *p* < 0.05. **(C)** Binding inhibition assay (BIA) using diluted Xanthan or proteinase K treated-EPS as competitors in the presence of calcium. The inhibition of the RapD-binding to the immobilized proteinase K treated-EPS was evaluated by preincubating the protein with different concentrations of EPS, Xanthan, or vehicle. Shown in the X-axis are the 10-fold dilutions of each respective competitor starting from 0.1 mg/ml. Asterisks indicate statistical differences between the 3 treatments at a 100-fold dilution of the inhibitory compound analyzed by one-way ANOVA with an α of 0.05 using Tukey post-test; *p* < 0.005. **(D)** RapD-EPS binding dynamics analyzed by BIA: monosaccharide competition. Binding inhibition assay preincubating recombinant RapD with increasing concentrations of glucose, galactose, or glucuronic acid ranging from 0 to 300 mM before exposure to fixed proteinase K treated-EPS. A representative experiment of three independent replicas measured in duplicate is shown. An asterisk indicates statistical differences between galactose and glucose or glucuronic acid at 300 mM analyzed by one-way ANOVA with an α of 0.05 and Tukey post-test; p < 0.05. **(E)** Competition between RapD and RapA2 for the binding to the EPS. The *Rhizobium*-EPS was pre-incubated with RapA2, BSA (0–10 μg/ml) or buffer (vehicle) before addition of RapD (5 μg/ml) (left). EPS was incubated at the same time with RapD (5 μg/ml) and increasing concentrations of RapA2 or BSA (0–10 μg/ml) (right).

Previous studies have shown that the Ra/CHDL domains of RapA2 confer the ability to bind the acidic polysaccharide either as CPS or in its released form (Abdian et al., 2013). The N-terminal domain of RapD consists of a Ra/CHDL domain which shares a 41% identity with the C-terminal Ra domain (Ra2) of RapA2 (Figure 1 and Supplementary Figure 6), suggesting that RapD would be able to bind the polysaccharide through this domain. In line with said hypothesis, we showed that protein-free-EPS also exerts a conformational change in RapD probably by means of direct interaction between the EPS and this domain. To assess whether RapD directly interacts with the EPS, we first analyzed by ELISA the presence of endogenous RapD associated with EPS preparations obtained from the extracellular medium of the *Rlv* wild type strain compared with those of *rapD* or secretion mutants (Figure 7A). The polysaccharides were immobilized on the wells and endogenous RapD was revealed with anti-RapD antibodies. The signal levels observed in the EPS preparation from the wild-type strain were significantly higher than those of the Δ*rapD*, Δ*rapD ΔrapA2* or *prsD* mutants, and a proteinase K-treated EPS, indicating that RapD is retained by the EPS. Interestingly, an increment of (30 ± 8) % in the EPS-associated RapD was consistently observed in the *rapA2* mutant (Figure 7A), suggesting that there is an interplay between both Rap(s) and their interaction with the EPS.

A direct ELISA assay was also performed using immobilized proteinase K-treated-EPS from *Rlv* 3841 wild type strain exposed to several concentrations of purified recombinant RapD ranging from 0.05 to 10 μg/ml in the presence of calcium. This analysis revealed a concentration-dependent binding of RapD with the EPS (Figure 7B). Surprisingly, under the assayed conditions, xanthan was also able to interact with recombinant RapD protein although to a lesser extent. This observation may be due to structural similarities between the EPS of *Rlv* and xanthan since both are anionic and are made up of repeating units of three sugars: two neutral and one anionic; glucose, glucuronic acid and galactose (in the case of EPS from *Rhizobium*) and glucose, galacturonic acid and mannose (in the case of xanthan) (Casas et al., 2000).

To further study the interaction of RapD with the EPS, a binding inhibition assay (BIA) using a modified ELISA technique was performed. Briefly, before incubation of recombinant RapD with immobilized-proteinase K treated EPS, binding inhibition was evaluated by adding several dilutions of proteinase K treated-EPS, xanthan, or vehicle (buffer), in the presence of calcium. Only the *Rlv* EPS (and not the xanthan) inhibited the binding of RapD to immobilized EPS in a concentration-dependent manner, suggesting that there is specific recognition of RapD for the *Rlv* EPS (Figure 7C).

As mentioned, the EPS of *Rlv* 3841 consists of octasaccharide repeating units containing glucose, glucuronic acid, and galactose (5:2:1). We have previously shown that the binding of RapA2 to the CPS/EPS was inhibited by glucuronic acid in the range of 20–100 mM (Abdian et al., 2013). To test whether RapD recognizes a particular monosaccharide on the EPS molecule, BIA analyses were carried out in the presence of free sugars and calcium. Results indicated that the RapD interaction with the EPS was partially inhibited in the presence of high concentrations of galactose in the mM range but not by glucose or glucuronic acid (Figure 7D), suggesting that the galactose might be implicated in the oligosaccharide structure recognized by RapD on the EPS molecule.

To investigate whether RapA2 can occupy RapD binding sites or recognize similar structures on the polysaccharide, a competition assay was performed preincubating the immobilized EPS with increasing concentrations of RapA2 or incubating simultaneously the immobilized EPS with RapD in the presence of increasing concentrations of RapA2. Under these conditions, no inhibition of the EPS-RapD binding was observed, suggesting that the sites recognized by these Rap(s) could be different (Figure 7E).

### EPS Profile: RapD and RapA2 Interplay

To evaluate whether RapD affects properties related to biofilm formation, diverse biofilm-associated phenotypes were analyzed in the *rapD* mutant and *rapD*-overexpressing strain. However, no obvious differences in macrocolony morphology, adhesion on polystyrene, swimming and swarming motilities, and EPS production were observed between the wild type strain and the Δ*rapD*,Δ*rapD/*pBBR*rapD*, and wild type/pBBR*rapD* strains grown in rich or Y-minimal media (Supplementary Tables 2, 3).

**FIGURE 8:**
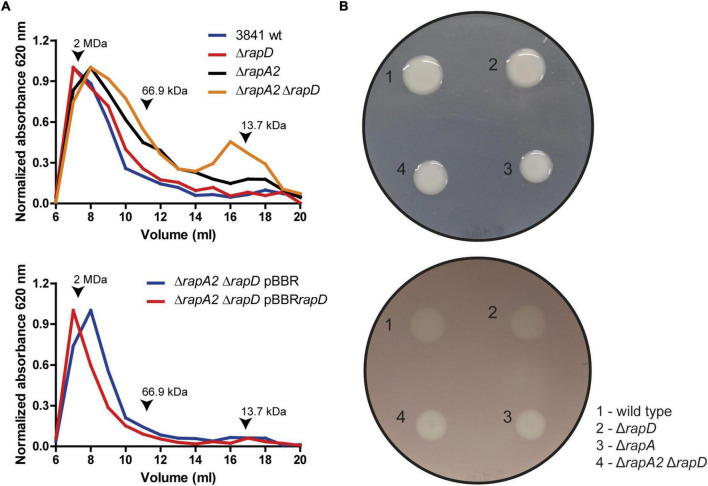
EPS analysis by size exclusion chromatography. **(A)** Upper panel: Molecular weight (MW) profiling of the EPS from wild type and derivative strains. Lower panel: Effect of RapD overexpression. Arrows indicate molecular weight markers. **(B)** Glycanase activity assay. Upper panel: The phenotypes of the colonies before revealing the glycanase assay are shown. Lower panel: The glycanase activity from each *Rlv* 3841 derivative strain is evidenced by a clearing (halo) under the colony after 5 days of incubation on Y mannitol CMC-containing plates.

As mentioned earlier, we have previously shown that RapA levels influence the EPS size distribution. To evaluate if the absence of RapD or the absence of both RapA and RapD modify the EPS profile, the EPS obtained from the extracellular medium of the wild type and isogenic mutants were analyzed by size exclusion chromatography (SEC), using a Superose 6 HR 10/30 column as described in Methods. This analysis showed an increased proportion of lower molecular weight species in comparison with the wild type strain both in the *rapD* and *rapA2* single mutants, which was even more evident in the *rapA2 rapD* double mutant (Figure 8A). We also studied the effect on the EPS profile of RapD overproduction in the strain that showed the most marked difference in size species (the *rapA2 rapD* double mutant). Overproduction of RapD restored the EPS profile to higher MW species (Figure 8A).

The differences in the molecular weights of the EPS species in the *rap* mutants may be due to a greater activity of EPS-glycanases from the Rap family, such as PlyB. It should be noted that the PlyA and PlyB glycanases are only active on the cell surface (Zorreguieta et al., 2000). Another possibility is that in the absence of RapA/RapD, the polysaccharide chains emerging from the cell surface are more susceptible to glycanase action. We evaluated glycanase activity using carboxymethyl cellulose (CMC) as a substrate model on a plate assay (Finnie et al., 1997). The extracellular β 1,4-glycanase activities were similar in the wild type and *rapD* mutant, showing only slightly increased activity in *rapA2* and *rapA2 rapD* mutants with no apparent differences between these two strains (Figure 8B). These results suggest that differences observed in the EPS-profiles of the wild type and *rapD* mutant or the *rapA2* and *rapA2 rapD* mutant cannot be accounted only for an increased glycanase activity in the extracellular medium.

Taken together, these observations suggest that the differences in the EPS profiles of the Rap mutant strains compared with those of the wild type strain may be due to the interaction of RapA/D proteins with the emerging polymer chains, which, in turn, would modulate the susceptibility of these molecules to Ply glycanases.

## Discussion

In this study, we showed that RapD is a calcium-dependent EPS-binding lectin that is co-secreted with the other Rap(s) and several proteins by the PrsDE TISS of *Rlv*. We observed that RapD was more actively secreted in Y-mannitol minimal medium than in a rich medium. This observation is interesting since copious amounts of EPS are produced in Y-mannitol in which *Rlv* forms structured biofilms (Russo et al., 2006; Vozza et al., 2016). In line with this observation, a previous transcriptomic analysis has shown that under sessile conditions, the *rapD* gene (named *rapB2*) of *Rhizobium etli* CFN42 was overexpressed when compared with *rapD* expression in the motile planktonic fraction of the culture (Reyes-Perez et al., 2016). Accordingly, we showed that in *Rlv* 3841, RapD is secreted in biofilms grown in Y-minimal medium. Besides, RapD is almost completely released into the extracellular medium, suggesting a different extracellular function from that of RapA proteins.

As described previously, upon secretion, a proportion of the RapA CPS/EPS-lectin is retained on the bacterial cell surface (Vozza et al., 2016). Immunofluorescence analysis showed that, under conditions of endogenous expression, numerous *Rlv* cells show RapA-signal at one cell pole but in several cells, the RapA signal resembles the capsule’s localization. Furthermore, under *rapA1* overexpressing conditions, most bacterial cells showed this pattern. This effect was explained by the lectin activity of RapA proteins toward the CPS (Vozza et al., 2016). On the other hand, two other Rap proteins, the PlyA and PlyB glycanases cleave the EPS and the CMC only when they are in contact with the bacterial surface. To note, although the PlyA protein appears to be retained on the cell surface, PlyB diffuses freely into the extracellular medium even though it would only be active on the cell surface (Zorreguieta et al., 2000). Thus, it is clear that some function(s) of the Rap(s) are associated with the cell surface, more precisely, with the CPS. One possibility is that a probable RapD function is related to the modulation of the EPS structure (rather than that of the CPS), modifying in some way some characteristics of the extracellular matrix. Another option is that the actions of the Rap(s) are performed sequentially; initially surface-associated RapA would exert its action on the CPS and the EPS chains. Later, RapD would act in a place more “detached” from the cell surface and mostly on the EPS. Interestingly, immunofluorescence of bacterial cells using anti-RapD antibodies showed few fluorescent signals of RapD at one pole of the cell surface, suggesting a transient polar localization of RapD. It was proposed that the CPS/EPS chains emerge initially from one bacterial end (Sherwood et al., 1984). It is tempting to hypothesize that all Rap proteins are secreted through the same pole as that where the CPS/EPS is initially produced, allowing a more efficient interaction of Rap(s) with the CPS or the EPS.

Studies by CD and fluorescent spectroscopy indicated that RapD exhibits a calcium-dependent β-structure that specifically binds the EPS of *Rlv*. Current models for secretion of RTX proteins by TISS indicate that these proteins are translocated through the three-component system in an unfolded state guided by a non-cleavable C-terminal secretion signal. The parallel β-roll structures bind extracellular calcium as they emerge from the cellular surface and direct protein conformation and translocation (Bumba et al., 2016; Motlova et al., 2020). The consolidation of RapD structure was observed in the millimolar range, which is consistent with the concentration of calcium found in the extracellular environment. As it was proposed for RapA2, in the cytoplasm, where the calcium concentration range is 0.1 – 2 μM (Watkins et al., 1995), RapD would have a molten globule-like structure that facilitates its direct translocation from the cytoplasm to the extracellular medium by the tripartite TISS. It is expected that RapD would acquire the β structure in the extracellular environment to achieve a productive interaction with the EPS.

RapA2 consists of two Ra/CHDL domains and has shown to be a flexible protein reliant on calcium for both secondary and possibly tertiary structures (Abdian et al., 2013). Likewise, RapD, which harbors a similar amino-terminal Ra/CHDL domain, exhibits conformational changes in the presence of calcium at a similar concentration range. However, far-UV CD spectra of RapD did not strictly resemble that of RapA2 since the latter only showed the consolidation of the negative band at ∼217 nm but does not show the calcium-dependent decrease in the 210–220 nm negative component (Abdian et al., 2013). This is probably due to the different behavior of the C -terminal regions of RapA2 and RapD. Another peculiarity was that the fluorescent spectrum of the single tryptophan present at the N-terminal region of the Apo-RapD shows a peak at ∼345–350 nm, which is indicative of a marginal exposition to the solvent. Moreover, upon addition of calcium, the peak center red-shifted to ∼355–360 nm indicating further exposure. However, the ellipticity increase and the red shift in the fluorescent spectrum occur at the same calcium concentrations indicating both changes occur simultaneously and that the increased interaction with the solvent might be a local event enhanced by the acquisition of secondary structure in response to calcium.

Analyses by size exclusion chromatography and static light scattering indicate that 1.25 mM of calcium induces oligomeric forms of RapD, likely dimmers, and trimmers. As mentioned before, RapA2 does not form oligomers in the presence of calcium, excluding for the function of this protein a cadherin-like mechanism (Abdian et al., 2013). In contrast, RapD forms oligomers in the presence of calcium, which seems to represent a distinctive characteristic. The hypothesis arises that the C-terminal region of RapD is responsible for multimerization and, therefore, for the protein behavior observed in the CD and fluorescence spectroscopy spectra. Extracellular, multimeric proteins have been shown to alter the biofilm structure and serve diverse functions. Most documented ones are usually high molecular weight structures (or amyloid fibers) that are in direct contact with the cell wall (Conrady et al., 2008; Taglialegna et al., 2016). There have been reports of lower molecular weight multimeric proteins that play different functions in biofilm matrix formation such as directing the EPS toward specific regions of the biofilm or mediating the tripartite cell-cell-EPS contact (Tielker et al., 2005; Giglio et al., 2013; Maestre-Reyna et al., 2013). In agreement with the second group of multimeric proteins, RapD constitutes one of the few examples of low molecular weight, multimeric-matrix-born proteins with an active role in matrix development. However, unlike their counterparts, there is no evidence that it remains tethered to the bacterial surface. It is possible that the formation of multimers of RapD provides an EPS-lectin multivalent character, which would favor intercatenary interactions between different EPS molecules for the development of a robust biofilm matrix. Further studies will be necessary to determine the RapD domain responsible for multimerization and the role of the multimer formation in RapD function.

We observed that in addition to the shift to a β-sheet structure induced by calcium, RapD undergoes further structural changes after adding the EPS. This observation suggests that RapD [and probably other Rap(s)] acquire its proper and functional folding in the presence of both calcium and the EPS. This could explain the reason why the PlyB glycanase diffuses to the extracellular media but the glycanase activity was only seen on the bacterial surface of EPS-producing bacteria (Zorreguieta et al., 2000). Thus, extracellular PlyB (already bound to calcium ions) could undergo additional conformational changes when interacting with some structure of the EPS, which, in turn, activates its glycanase activity on the EPS chains. Therefore, in our model, the Rap proteins are secreted in an unfolded state due to low cytosolic calcium concentrations. As soon as the Rap protein reaches the extracellular milieu, it binds calcium ions, folds properly, and eventually forms multimeric structures, enabling the interaction of Rap with some emerging EPS/CPS molecules. Finally, the interaction of Rap with some structure of the polysaccharide will make the protein fully functional.

Mechanisms for interactions of polysaccharides with other molecules or structures vary in nature, being either through electrostatic charges or through defined binding sites on the polysaccharide (Tielen et al., 2013; Reichhardt et al., 2018; Passos, da Silva et al., 2019). Within the same species both mechanisms can coexist, such is the case for *P. aeruginosa*’s lectins LecB and CdrA, both of which bind Psl polysaccharide (Borlee et al., 2010; Passos, da Silva et al., 2019). In this work, we demonstrated the specific binding of RapD to the EPS. This binding was partially inhibited by the addition of galactose, in the millimolar range, suggesting that galactose would be part of the EPS structure recognized by RapD, or that it could bind an oligosaccharide containing galactose. Interestingly, we have previously shown that the interaction of RapA2 with the EPS is blocked by glucuronic acid, also in the millimolar range (Abdian et al., 2013). It is worth mentioning that in the biological context, the avidity of the Rap lectin towards the EPS is likely to depend on a combination of more than one sugar and/or glycosidic linkages. Besides, although both RapA2 and RapD bind the EPS, the particular structure of the polysaccharide recognized by these Rap(s) could be different. In line with this idea, competition experiments between RapA2 and RapD for the EPS binding suggest that these proteins appear not to share the same binding site on the EPS. Further studies will be necessary to elucidate the precise EPS/CPS epitopes recognized by the Rap(s).

Unlike the *rapA2* mutant, the *rapD* mutant did not display any obvious phenotype related to adhesion to surfaces or interactions between bacterial cells. The possibility exists that the profuse amounts of EPS produced by *Rlv* masks some structural or cell-cell alterations within the biofilm of the mutant (Reichhardt and Parsek, 2019). The absence of RapD secretion, however, produced slight changes in the EPS profiles obtained by size exclusion chromatography while the lack of RapA2 resulted in a major effect, specifically towards species of lower molecular weights. These changes in EPS sizes could be, in part, to an augmented glycanase activity in these genetic backgrounds, suggesting interplay in the regulation of the expression/secretion of Rap proteins and matrix assembly. Interestingly, the absence of both Rap(s) resulted in size distribution of EPS molecules of even smaller sizes compared to those of the single mutants while showing the same glycanase activity levels. One hypothesis is that *Rlv* matrix development is a multi-step process carried out and coordinated by several extracellular factors including RapA2, RapD, and Ply glycanases, in which the absence of the first two results in a deregulated processing, leading to a higher proportion of EPS of smaller sizes.

To our knowledge, secretion of various structural and functionally related proteins with lectin activity towards the polysaccharide produced by the same bacteria has not been documented. There are few reports of extracellular proteins interacting, altering, or driving matrix architecture in other bacteria (Reichhardt and Parsek, 2019; Domenech and Garcia, 2020). In *Vibrio cholerae*, matrix proteins follow a strict spatial distribution, which in turn helps them interact with the polysaccharide (VPS) (Berk et al., 2012). Alongside previous findings (Abdian et al., 2013; Vozza et al., 2016), we showed that despite strong similarities between the Ra-lectin domains, Rap proteins appear to bind different structures on the EPS and play different roles during biofilm formation in *Rlv*. We propose that matrix development in *Rlv* is a multistep process regulated not only temporally but also spatially as seen by the different niches of the Rap proteins.

## Data Availability Statement

The original contributions presented in this study are included in the article/Supplementary Material, further inquiries can be directed to the corresponding author/s.

## Ethics Statement

The animal procedures and protocols used to generate a polyclonal antisera against RapD were reviewed and approved by Comité de Bioética Fundación Instituto Leloir Protocol number: 2009 01 BFIL.

## Author Contributions

JT: substantial contributions to the conception and design of the work, acquisition, analysis, and interpretation of data for the work, drafting and writing the work, and final approval of the version to be published. LR: acquisition, analysis, and interpretation of data for the work. LA: analysis and interpretation of data for the work, drafting and writing the work, and final approval of the version to be published. DR and AZ: substantial contributions to the conception and design of the work, analysis and interpretation of data for the work, drafting and writing the work, and final approval of the version to be published. All authors contributed to the article and approved the submitted version.

## Conflict of Interest

The authors declare that the research was conducted in the absence of any commercial or financial relationships that could be construed as a potential conflict of interest.

## Publisher’s Note

All claims expressed in this article are solely those of the authors and do not necessarily represent those of their affiliated organizations, or those of the publisher, the editors and the reviewers. Any product that may be evaluated in this article, or claim that may be made by its manufacturer, is not guaranteed or endorsed by the publisher.
