# Supplementary material for: RapD Is a Multimeric Calcium-Binding Protein That Interacts With the Rhizobium leguminosarum Biofilm Exopolysaccharide, Influencing the Polymer Lengths
Source: Front Microbiol. 2022 Jul 6;13:895526. doi: 10.3389/fmicb.2022.895526 (PMC9298526; doi:10.3389/fmicb.2022.895526)
Supplement: Supplementary file 1 [file Table_1.DOCX]

**Table S1**: NCBI accession codes for proteins mentioned in this work

| Locus tag | Description | Accession number |
| --- | --- | --- |
| RL3024  Q1MEW2 | PlyB | [CAK08512](https://www.ncbi.nlm.nih.gov/protein/CAK08512) |
| RL0790  Q1ML69 | Putative calcium binding RTX Zn-metalloprotease | [CAK06284](https://www.ncbi.nlm.nih.gov/protein/CAK06284) |
| pRL90140  Q1M8U8 | Beta helix domain-containing protein | [CAK03856](https://www.ncbi.nlm.nih.gov/protein/CAK03856) |
| RL2961  Q1MF23 | Putative calcium-binding cadherin-like RTX protein | [CAK08451](https://www.ncbi.nlm.nih.gov/protein/CAK08451) |
| pRL100175  Q1M7X8 | NodO | \|  \| [CAK10399](https://www.ncbi.nlm.nih.gov/protein/CAK10399) \| \| --- \| --- \| |
| pRL100309  Q1M7J7 | Putative calcium-binding cadherin-like RTX protein | \|  \| [CAK10534](https://www.ncbi.nlm.nih.gov/protein/CAK10534) \| \| --- \| --- \| |
| pRL100451  Q1M755 | RapA2 | CAK10677 |
| RL3659  Q1MD30 | PlyA | [CAK09148](https://www.ncbi.nlm.nih.gov/protein/CAK09148) |
| RL2412  Q1MGL7 | Putative calcium binding RTX protein | [CAK07902](https://www.ncbi.nlm.nih.gov/protein/CAK07902) |
| RL2702  Q1MFT2 | RapD | CAK08191 |
| RL3023  Q1MEW3 | PlyC | CAK08511 |
| RL3073  Q1MER4 | RapC (C terminus ORF) | [CAK08561](https://www.ncbi.nlm.nih.gov/protein/CAK08561) |
| RL3911  Q1MCD0 | RapB | [CAK09401](https://www.ncbi.nlm.nih.gov/protein/CAK09401) |
| RL1580  Q1MIY5 | NDK – nucleoside diphosphate kinase | [CAK07075](https://www.ncbi.nlm.nih.gov/protein/CAK07075) |
