# Supplementary material for: RapD Is a Multimeric Calcium-Binding Protein That Interacts With the Rhizobium leguminosarum Biofilm Exopolysaccharide, Influencing the Polymer Lengths
Source: Front Microbiol. 2022 Jul 6;13:895526. doi: 10.3389/fmicb.2022.895526 (PMC9298526; doi:10.3389/fmicb.2022.895526)
Supplement: Supplementary file 3 [file Table_3.docx]

Supplementary table 3: Biofilm related phenotypes for *Rlv* 3841 and derivative strains carrying pBBR/pBBR*rapD* in TY and Y mannitol medium. Evaluated phenotypes include macrocolony morphology, adhesion of bacterial biomass to polystyrene, swimming and swarming motilities and EPS production. All measurements were done according to prior works (Russo et al. 2006, Vozza – Abdian et al. 2016, Sorroche et al. 2018).

|  |  | Macrocolony phenotype | Adhesion to polystyrene (relative to wt) | Swimming motility | Swarming motility | EPS production (mg/100 ml culture) |
| --- | --- | --- | --- | --- | --- | --- |
| TY | wt/pBBR | Mucoid, brilliant and convex shaped colony | 113.70 ± 23.3 | 1.83 ± 0.30 | ND | ND |
|  | wt/pBBR*rapD* | Mucoid, brilliant and convex shaped colony | 117.79 ± 24.2 | 1.76 ± 0.18 | ND | ND |
|  | *ΔrapD*/pBBR | Mucoid, brilliant and convex shaped colony | ND | 1.70 ± 0.10 | 0.93 ± 0.03 | ND |
|  | *ΔrapD*/pBBR*rapD* | Mucoid, brilliant and convex shaped colony | ND | 1.46 ± 0.32 | 0.95 ± 0.05 | ND |
| Y | wt/pBBR | Mucoid, brilliant and convex shaped colony | 62.16 ± 8.40 | 1.50 ± 0.12 | ND | 110 |
|  | wt/pBBR*rapD* | Mucoid, brilliant and convex shaped colony | 71.34 ± 24 | 1.26 ± 0.14 | ND | 99.7 |
|  | *ΔrapD*/pBBR | Mucoid, brilliant and convex shaped colony | 125.04 ± 54.4 | 1.74 ± 0.12 | 1.57 ± 0.06 | 115.7 |
|  | *ΔrapD*/pBBR*rapD* | Mucoid, brilliant and convex shaped colony | 108.4 ± 23.6 | 1.48 ± 0.14 | 1.56 ± 0.01 | 108.7 |
