# Supplementary figures and images for: RapD Is a Multimeric Calcium-Binding Protein That Interacts With the Rhizobium leguminosarum Biofilm Exopolysaccharide, Influencing the Polymer Lengths
Source: Front Microbiol. 2022 Jul 6;13:895526. doi: 10.3389/fmicb.2022.895526 (PMC9298526; doi:10.3389/fmicb.2022.895526)

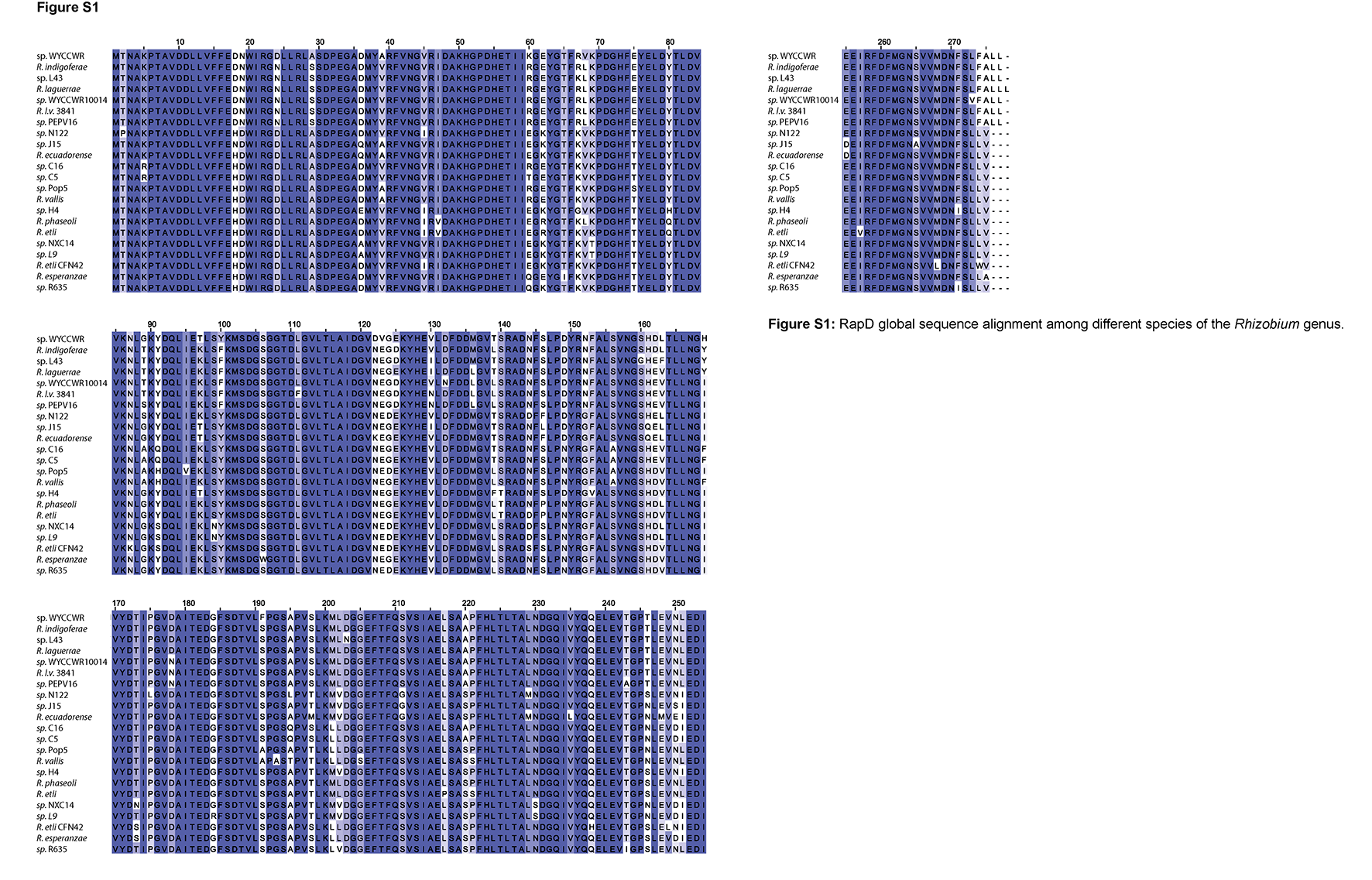

Supplement: Supplementary file 4 [file Image_1.TIF]

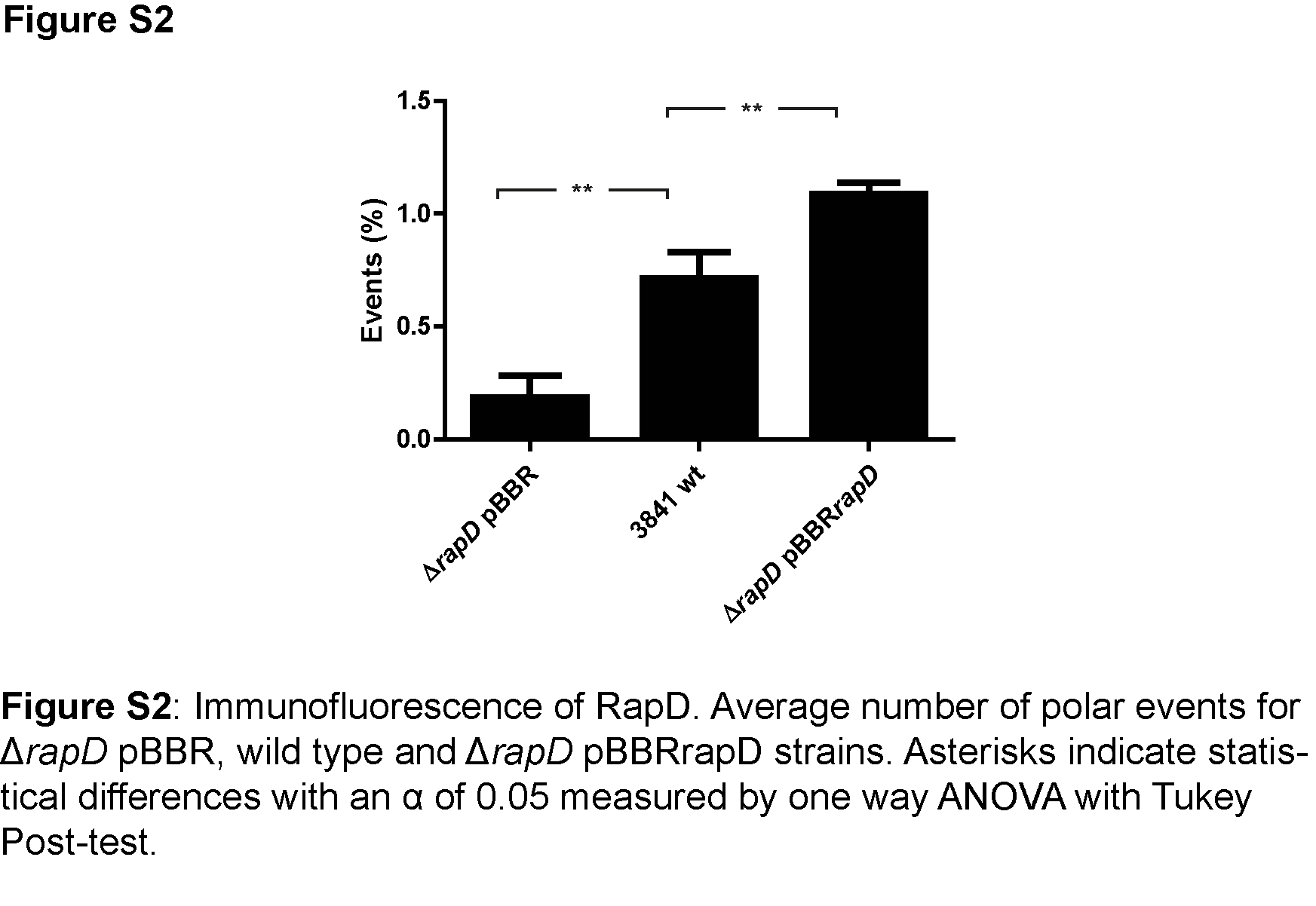

Supplement: Supplementary file 5 [file Image_2.TIF]

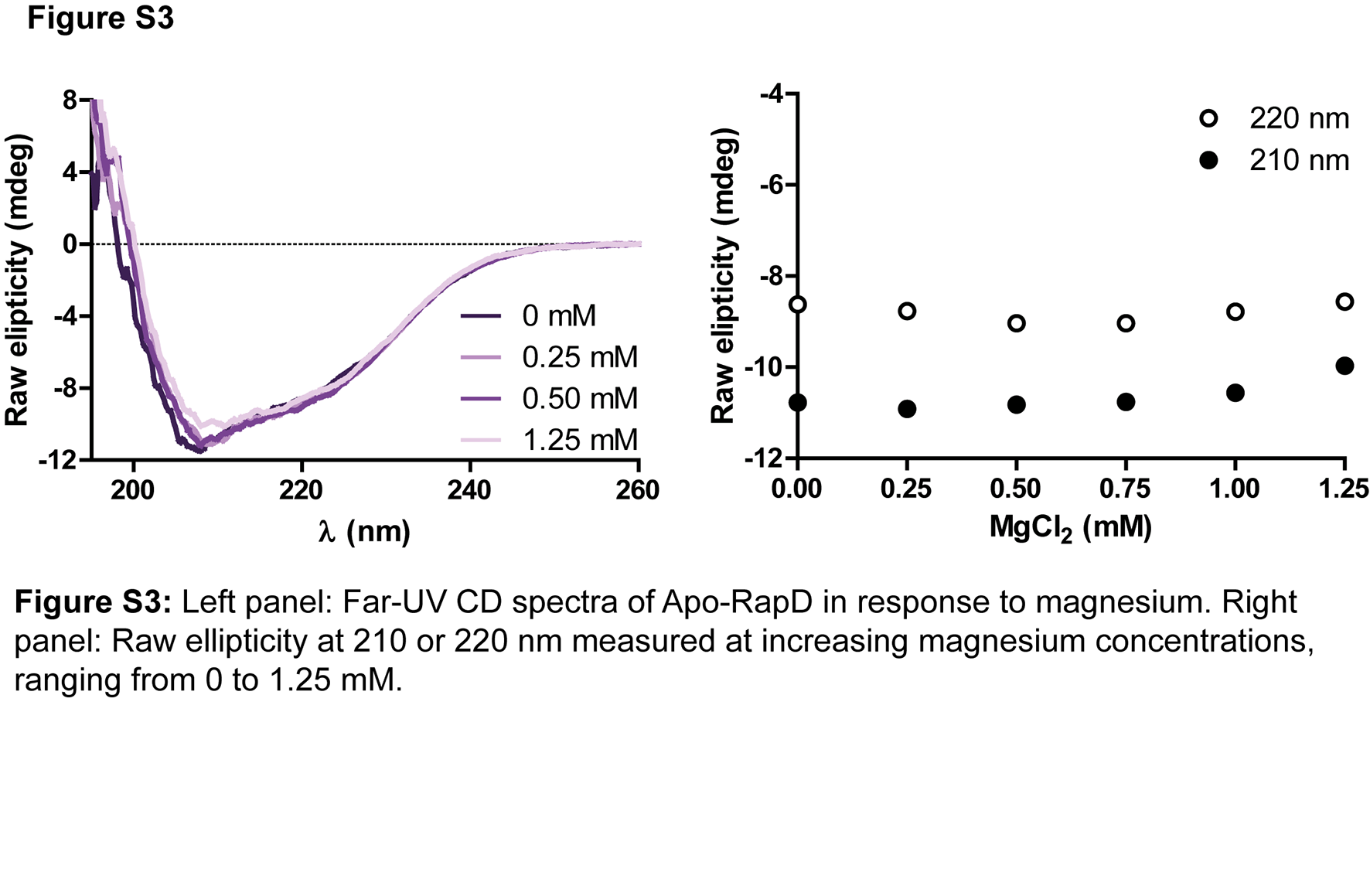

Supplement: Supplementary file 6 [file Image_3.tif]

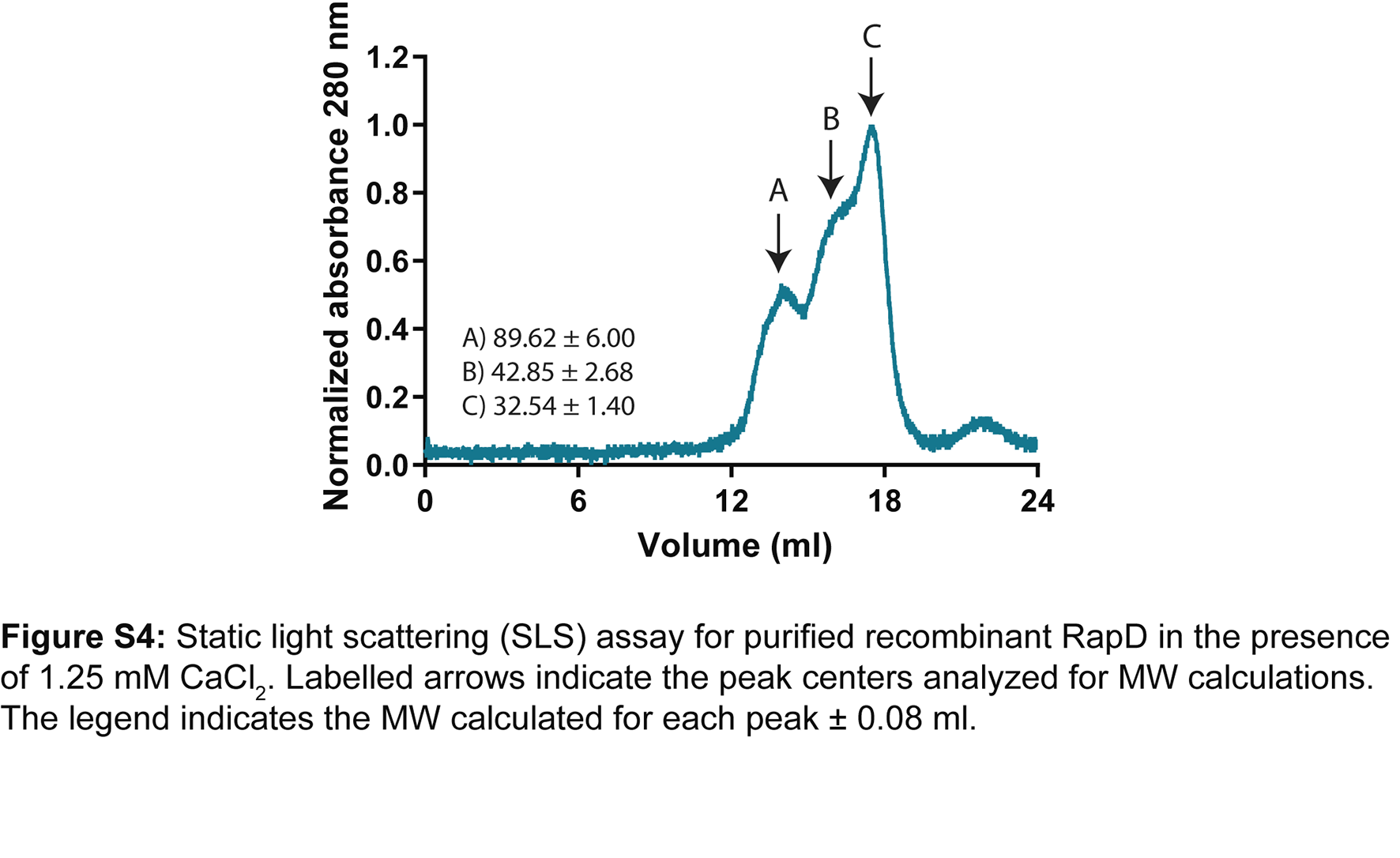

Supplement: Supplementary file 7 [file Image_4.tif]

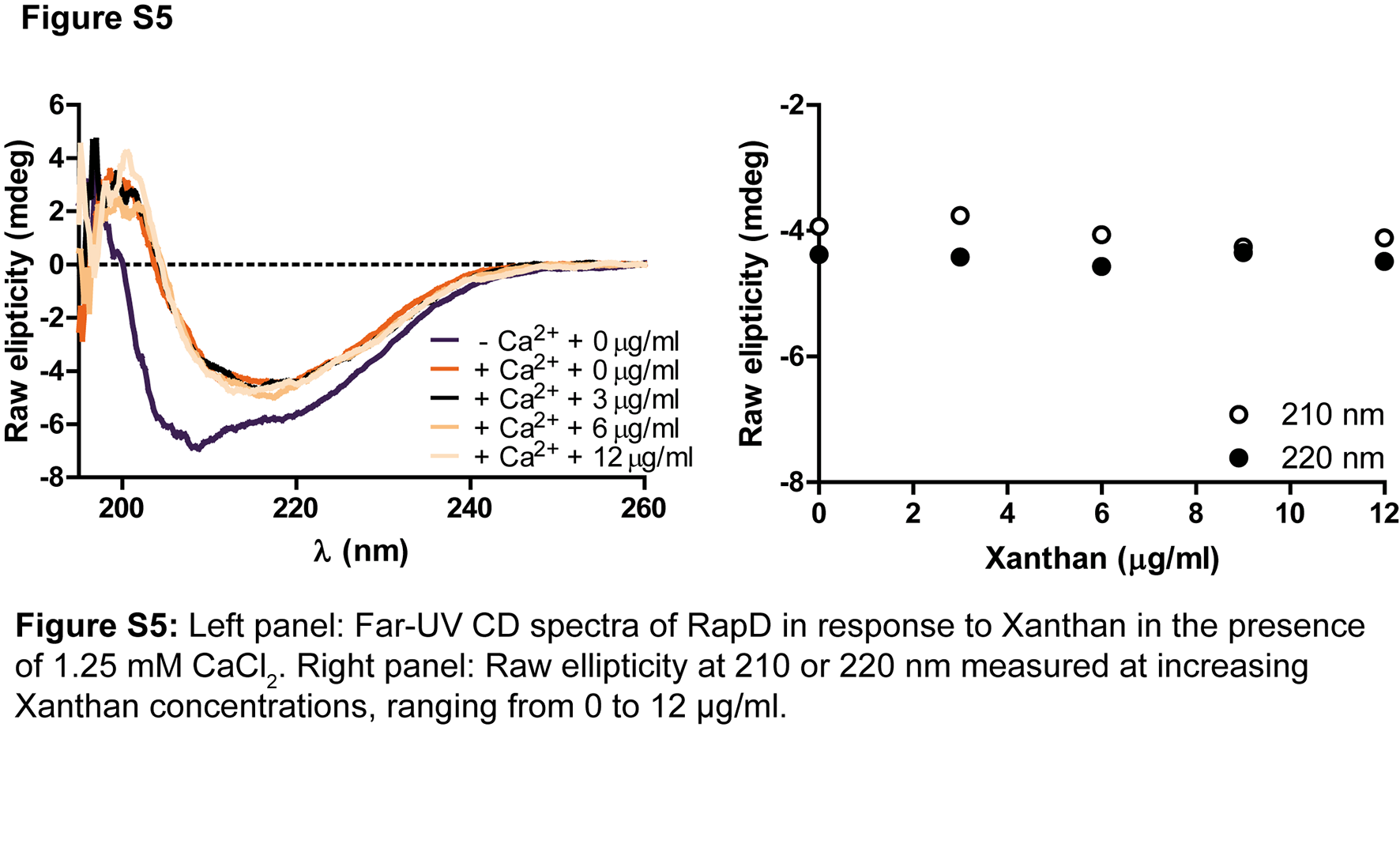

Supplement: Supplementary file 8 [file Image_5.tif]

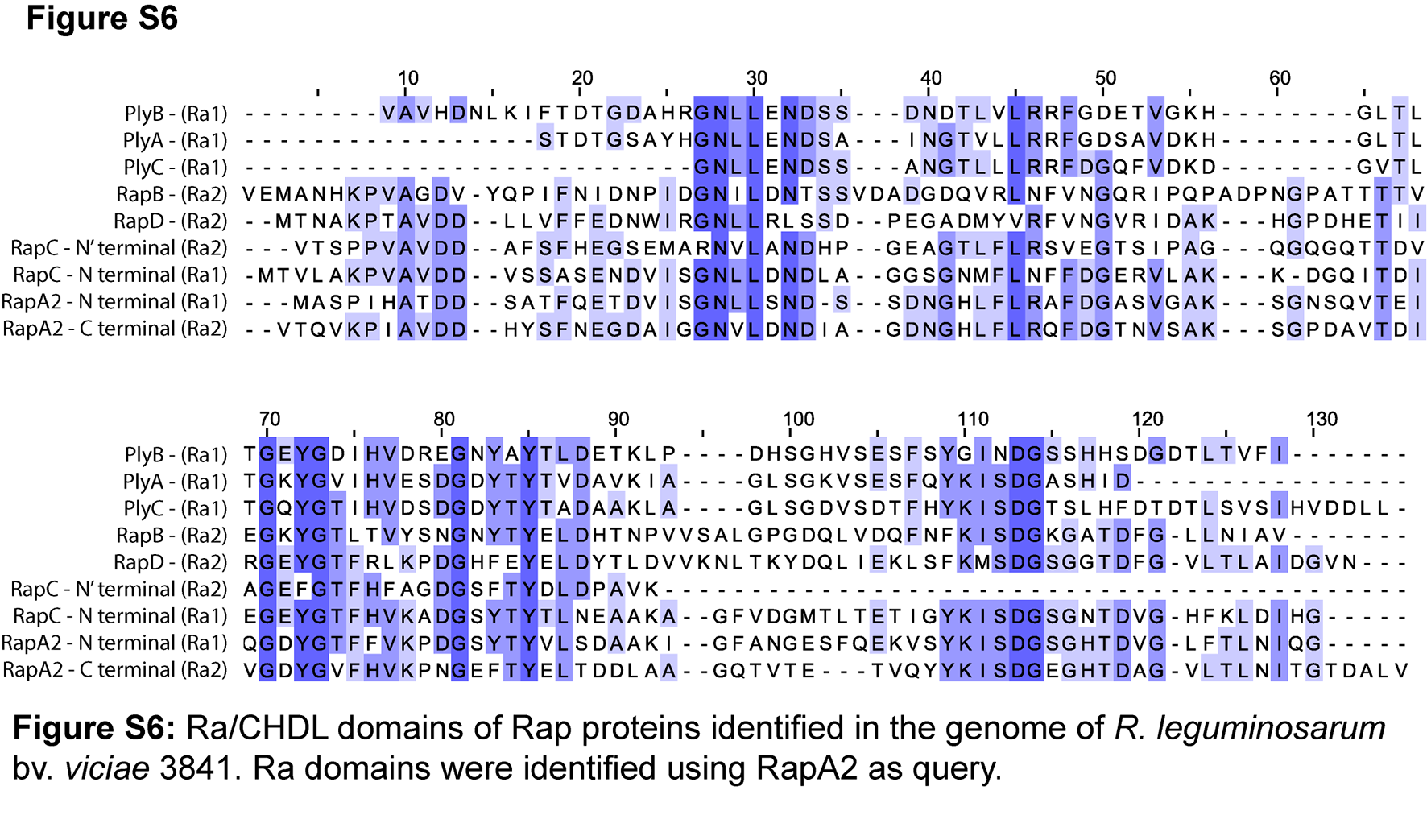

Supplement: Supplementary file 9 [file Image_6.tif]
